# Prodromal neuroinvasion of pathological α-synuclein in brainstem reticular nuclei and white matter lesions in a model of α-synucleinopathy

**DOI:** 10.1093/braincomms/fcab104

**Published:** 2021-05-14

**Authors:** Nelson Ferreira, Mette Richner, Amelia van der Laan, Ida Bergholdt Jul Christiansen, Christian B Vægter, Jens R Nyengaard, Glenda M Halliday, Joachim Weis, Benoit I Giasson, Ian R Mackenzie, Poul H Jensen, Asad Jan

**Affiliations:** 1 Department of Biomedicine, Danish Research Institute of Translational Neuroscience (DANDRITE), Aarhus University, DK-8000 Aarhus C, Denmark; 2 DANDRITE, Nordic-EMBL Partnership for Molecular Medicine, Aarhus University, DK-8000 Aarhus C, Denmark; 3 Core Center for Molecular Morphology, Section for Stereology and Microscopy, Department of Clinical Medicine, Aarhus University, DK-8200 Aarhus N, Denmark; 4 Brain and Mind Centre and Faculty of Medicine and Health, School of Medical Sciences, University of Sydney and Neuroscience Research Australia, Sydney 2006, Australia; 5 Institute of Neuropathology, RWTH Aachen University Hospital, Aachen 52074, Germany; 6 Department of Neuroscience, University of Florida, Gainesville, FL 3261, USA; 7 Department of Pathology and Laboratory Medicine, University of British Columbia, Vancouver, BC V6T2B5, Canada

**Keywords:** Parkinson disease, α, -synuclein, lewy pathology, prion-like, M83 transgenic mice

## Abstract

Neuropathological observations in neurodegenerative synucleinopathies, including Parkinson disease, implicate a pathological role of α-synuclein accumulation in extranigral sites during the prodromal phase of the disease. In a transgenic mouse model of peripheral-to-central neuroinvasion and propagation of α-synuclein pathology (via hindlimb intramuscular inoculation with exogenous fibrillar α-synuclein: the M83 line, expressing the mutant human Ala53Thr α-synuclein), we studied the development and early-stage progression of α-synuclein pathology in the CNS of non-symptomatic (i.e. freely mobile) mice. By immunohistochemical analyses of phosphroylated α-synuclein on serine residue 129 (p-S129), our data indicate that the incipient stage of pathological α-synuclein propagation could be categorized in distinct phases: (i) initiation phase, whereby α-synuclein fibrillar inoculum induced pathological lesions in pools of premotor and motor neurons of the lumbar spinal cord, as early as 14 days post-inoculation; (ii) early central phase, whereby incipient α-synuclein pathology was predominantly detected in the reticular nuclei of the brainstem; and (iii) late central phase, characterized by additional sites of lesions in the brain including vestibular nuclei, deep cerebellar nuclei and primary motor cortex, with coincidental emergence of a sensorimotor deficit (mild degree of hindlimb clasping). Intriguingly, we also detected progressive α-synuclein pathology in premotor and motor neurons in the thoracic spinal cord, which does not directly innervate the hindlimb, as well as in the oligodendroglia within the white matter tracts of the CNS during this prodromal phase. Collectively, our data provide crucial insights into the spatiotemporal propagation of α-synuclein pathology in the nervous system of this rodent model of α-synucleinopathy following origin in periphery, and present a neuropathological context for the progression from pre-symptomatic stage to an early deficit in sensorimotor coordination. These findings also hint towards a therapeutic window for targeting the early stages of α-synuclein pathology progression in this model, and potentially facilitate the discovery of mechanisms relevant to α-synuclein proteinopathies. In a rodent model of synucleinopathy, Ferreira et al., delineate the spatiotemporal progression of incipient α-synuclein pathology (of peripheral origin) in the CNS. The authors show early affection of brainstem reticular nuclei in non-paralyzed mice, and pathological white matter lesions in relation to the neuronal pathology.

## Graphical Abstract

**Graphical Abstract:**
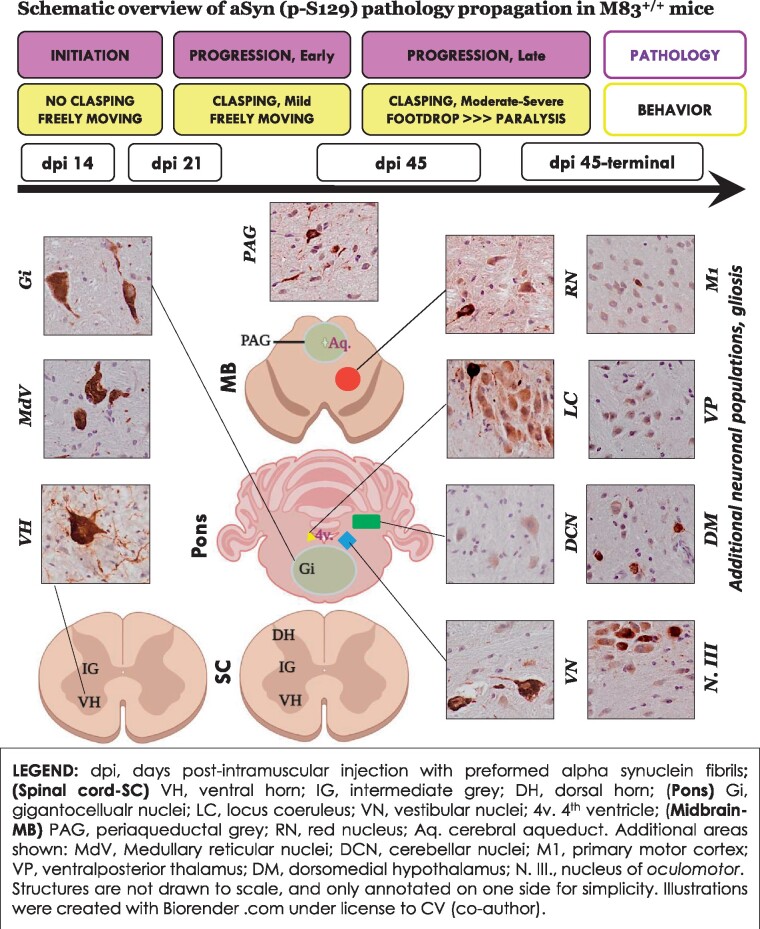

## Introduction

Pathological accumulation of α-synuclein (aSyn; gene symbol *SNCA*) is a hallmark feature of the neurodegenerative diseases termed synucleinopathies, that include Parkinson disease (PD), Multiple system atrophy (MSA) and Dementia with Lewy bodies (DLB).^1^^,^^2^ Among these disorders, idiopathic PD remains the most common cause of motor disability, and is clinically characterized by resting tremor, rigidity, difficulty in movement initiation and postural instability.^3^^,^^4^ The loss of dopamine producing neurons in the midbrain substantia nigra (SN)-pars compacta is considered to be a major factor in the aetiology of tremor and movement disability associated with PD. In addition, neuronal dysfunction and/or demise in extranigral motor control areas compound the postural instability and gait apraxia.^3–5^ While the majority of cases (>85%) are diagnosed with idiopathic (late-onset) PD, missense mutations in *SNCA* or multiplications in the *SNCA* gene locus cause rare inherited disorder in a subset of the patients.^4^^,^^6^^,^^7^

Neuropathological observations implicate a pathological role of proteinaceous inclusions containing aggregated aSyn, found in neuronal somata and in dystrophic neurites [identified as Lewy bodies (LBs) and Lewy neurites (LNs), respectively].^4^^,^^7^^,^^8^ Biochemically, ∼90% of aggregated aSyn in these lesions is phosphorylated at the serine residue 129 (S129)^9^^,^^10^ and is considered a reliable marker of LB pathology.^1^^,^^2^ aSyn LB pathology and resulting neuronal loss in PD is not random, such that specific grey matter nuclei and neuronal populations are preferentially affected in the early stages, while some brain regions are relatively spared.^5^^,^^8^^,^^11^ Recent studies in cell culture and animal models suggest that aSyn misfolding and aggregation can propagate between cells, including neurons [reviewed in detail elsewhere^11^^,^^12^]. The insights gained from these studies have led to the emergence of ‘the prion hypothesis of aSyn’. According to this hypothesis, certain conformational states of aSyn—termed ‘seeds’—act as templates for native aSyn aggregation, and propagate through the connected neuroanatomical tracts in the CNS.^8^^,^^11^^,^^12^ Accordingly, direct inoculation of exogenous aSyn seeds into select brain regions in rodents, or in a peripheral location (e.g. muscle), induces various degrees of PD-like aSyn pathology in the CNS.^11^

The transgenic M83^+/+^ mice express mutant human Ala53Thr (A53T) aSyn driven by the mouse prion promoter, and develop spontaneous Lewy related aSyn pathology after 7 months of age (median age of onset, ∼12 months), which coincides with severe motor impairment.^13^^,^^14^ This moribund phenotype is significantly exacerbated by the exogenous inoculation of pre-formed fibrillar (PFF) aSyn, delivered intramuscularly in the hindlimb, in younger (2–3 months old) M83^+/+^ mice. Accordingly, pathological aSyn of peripheral origin induces an aggressive form of motor disability, which appears around 50–70 days post-injection, and leads to a drastic reduction in survival within 2–3 weeks of onset.^13^ Neuropathologically, there is widespread accumulation of phosphorylated aSyn (p-aSyn, S129) in the CNS, with predominant affection of lumbar spinal cord and brainstem regions, and relative paucity in the forebrain areas.^13^^,^^14^ This experimental model has certain limitations to be considered as a *bona fide* paradigm for CNS synucleinopathies, i.e. a peripheral route of aSyn pathology induction and initial propagation in neuroanatomical tracts not significantly implicated in PD (also see Discussion). Nevertheless, several laboratories, including our own, have reproducibly used this model to investigate the biochemical and behavioural sequelae of aSyn trans-synaptic propagation that may underlie neuronal dysfunction and toxicity associated with aSyn accumulation in the CNS.^13^^,^^15–18^

Herein, we describe the early stages of aSyn pathology in the CNS following intramuscular PFF aSyn inoculation in homozygous M83^+/+^ mice, and report that a significant number of neuronal populations were affected long before any visible defects in sensorimotor behaviour became manifest. In this prodromal phase, our data revealed incipient aSyn pathology (detected by p-aSyn, S129 immunostaining) within pools of premotor and motor neurons in the spinal cord, and subsequent propagation into the reticular nuclei of brainstem, that are known to control the activity of the somatomotor system. Furthermore, we also detected p-aSyn (S129) accumulation in the oligodendroglia within the CNS, both in the vicinity of neuronal populations affected by aSyn pathology as well as within the large white matter tracts of the somatomotor system. Incorporating these observations and predecessor studies in M83^+/+^ mice,^13^^,^^14^ we propose a spatiotemporal paradigm highlighting salient features of CNS aSyn pathology in relation to the presymptomatic and moribund stage in this experimental model of synucleinopathy (see Graphical abstract). We anticipate that this spatiotemporal paradigm will serve as a valuable reference in studying the mechanisms in trans-synaptic pathological aSyn propagation, and in characterising the therapeutic strategies’ mode of action in prevention and/or disease modification.

## Materials and methods

### Generation and characterization of mouse aSyn fibrils

Mouse aSyn fibrils (PFF) were prepared and characterized *in vitro*, essentially as described.^15^ Briefly, full length recombinant (wild type) mouse αSyn was expressed in BL21(DE3) competent cells and purified using reverse phase chromatography.^19^ To generate PFF aSyn, purified recombinant mouse α-Syn (5 mg/ml) was incubated at 37°C in phosphate buffered saline (PBS) pH 7.4 with continuous shaking at 1050 r.p.m. in a tabletop microtubes shaker (Eppendorf). The PFF were collected by centrifugation (15 600 *g* at 25°C for 30 min), and then re-suspended in PBS, Protein concentration was determined by the BCA assay and a stock solution consisting of 2 mg/ml protein was prepared (in PBS). Subsequently, PFF were sonicated for 20 min using a Branson 250 Sonifier at 30% intensity, and were aliquoted and frozen at −80°C until further use. The purity of these fibrillar preparations, their biophysical characterization and biological activity is described elsewhere.^15^

### Animal studies

#### Hindlimb intramuscular aSyn inoculation

Transgenic M83^+/+^ mice [B6; C3-Tg(Prnp-SNCA*A53T)83Vle/J]^14^ were housed at the Bartholin animal facility at Aarhus University in accordance with Danish regulations and the European Communities Council Directive for laboratory animals (license # 2017–15-0201–01203 issued to PHJ, co-author). The animals were housed under a 12 h light/dark cycle and fed with regular chow diet *ad libitum*. Homozygous M83^+/+^ mice (2–3 months old, *n* = 10/group) were bilaterally inoculated with a single injection (5 µl) of recombinant mouse aSyn PFF into the hindlimb biceps femoris (using a 10-μl Hamilton syringe with a 25-gauge needle) as described previously.^15^^,^^16^ Control group (*n* = 10, M83^+/+^) were injected with PBS (5 µl, bilaterally). The experiments included animals of both male and female sex.

#### Hindlimb clasping

Assessment of hindlimb clasping behaviour was performed by a modified tail suspension test.^15^^,^^16^^,^^20^ Freely moving, non-anaesthesized, M83^+/+^ mice were held by the tail and lifted in air for 10 seconds. Severity of clasping was scored on a scale of 0–4, as follows: (i) Score 0, No clasping (both hindlimbs were consistently splayed outwards, away from the abdomen); NOTE: To avoid ambiguity (false positive assessment), mice exhibiting unilateral hindlimb retraction towards the abdomen for less than 50% of the time were also scored 0 (i.e. clasping not established); (ii) Score 1, Mild clasping (one hindlimb was retracted towards the abdomen for more than 50% of the time suspended); (iii) Score 2, Moderate clasping (both hindlimbs were partially retracted towards the abdomen for more than 50% of the time suspended); and (iv) Score 3, severe clasping (both hindlimbs were entirely retracted and touching the abdomen for more than 50% of the time suspended).

#### Immunohistochemistry and immunofluorescence on murine tissues

Immunohistochemistry (IHC) or immunofluorescence (IF) on 10µm thick sections from formalin fixed paraffin embedded tissue was performed after deparaffinization and antigen retrieval in citrate buffer pH-6.0. Following primary antibodies were employed (and are also indicated in the relevant figure legends): phospho-S129 aSyn antibodies (mouse mAb 11A5, kind gift to PHJ by Imago Pharmaceuticals^21^– 1:1000; or rabbit mAb D1R1R, Cell Signaling #23706– 1:500; or rabbit mAb EP1536Y Abcam, #ab51253– 1:400), Neuronal nuclei marker (NeuN, mouse mAb A60, Millipore #MAB377- 1:500), astroglial marker, glial fibrillary acidic protein antibodies (GFAP, rabbit mAb D1F4Q, Cell Signaling #12389– 1:200; or chicken polyclonal, Abcam #4674– 1:200), phagocyte marker CD68 (LAMP4, rat mAb FA-11, Novus Biologicals #NBP2-33337– 1:200), Tubulin Polymerization Promoting Protein/p25 (Rabbit polyclonal^19^– 1:100) and Sequestosome 1/p62 (mouse mAb Abcam #ab56416– 1:500). For IHC, DAB (3,3'-diaminobenzidine) chromogen detection was performed following prior incubation with biotin conjugated secondary antibody (anti-mouse, Sigma #B7264– 1:100) and Extra-Avidin peroxidise (Sigma #E2886– 1:200). Sections were counterstained with haematoxylin (Vector Labs, #H-3401). For double and triple IF co-detection, Alexa-Fluor fluorophore conjugated secondary antibodies (Thermo Fisher) were used (AlexaFluor488, AlexaFluor568, AlexaFluor594 or AlexaFluor647, 1:1000). High resolution views were obtained using Olympus VS120 digital slide scanner (equipped for brightfield scanning and fluorescence single-band emitters for Hoechst, FITC, Cy3, Cy5 and Cy7), and magnified views (5×, 10×, 20× and 40×) were extracted using OlyVia software (Olympus). Alternatively, IF images were acquired using a Zeiss observer inverted microscope equipped with colibri 7 LED illumination, and operated using Zen (Zeiss) software.

#### Neuroanatomical topography

Panoramic images from the digital slide scans were mapped onto Mouse Brain Atlas (Paxinos and Franklin's The Mouse Brain in Stereotaxic Coordinates, Elsevier Publishing, Fourth Edition).^22^ Information about neuroanatomical tracts and nuclei in mouse CNS was primarily derived from The Mouse Nervous System (Elsevier Publishing, First Edition).^23^

### Human tissue processing and IHC

IHC detection of p-aSyn (S129) was performed on post-mortem human brain specimen using the following antibodies: Elan, mouse mAb (kind gift to the co-author GMH by Elan Pharmaceuticals^24^) or 81A, mouse mAb (EMD Millipore #MABN826) as described below.

#### IHC using Elan p-aSyn (S129) antibody

IHC data from post-mortem control, MSA and PD cases (Supplementary Table S1) using the Elan p-aSyn (p-S129) antibody were provided by GMH (co-author), after appropriate consent and ethics approval through the Australian Brain Donor Programme. Formalin-fixed paraffin-embedded tissue sections of the pons and midbrain from pathologically confirmed PD, MSA and neurological and neuropathological control cases were used. Demographic variables for the cohorts are presented in Supplementary Table S1. For IHC, routine immunoperoxidase staining of 10 µm paraffin-embedded sections was performed with phosphorylation-dependent antibody directed against amino acids 124–134 specific for p-aSyn (S129), provided by Elan Pharmaceuticals. Sections were pretreated with citrate buffer for microwave antigen retrieval containing formic acid, 5% hydrogen peroxide/50% ethanol blocking solution, and 10% normal horse serum blocking solution prior to overnight incubation in the primary antibody (p-aSyn, S129; 1:10 000). Biotinylated secondary antibodies and ABC tertiary kit were applied (Vector), followed by visualisation with 3,3′-diaminobenzidine (Sigma). Sections were counterstained with 0.5% cresyl violet prior to coverslipping with DPX mounting medium (Sigma). A negative (primary antibody omitted) and a positive (human DLB hippocampus tissue section containing LBs and LNs) control were included in each experiment to ensure specificity of the IHC. Immunostained sections were evaluated on a brightfield microscope (Zeiss AxioSkop) and photographed using a computerised image software system (Zeiss Axiocam HRC digital camera and Axiovision software version 4.6.3). Photomicrographs were taken at 100× magnification in three random fields and representative photomicrographs are illustrated.

#### IHC using 81A p-aSyn (S129) antibody

IHC data from post-mortem control and PD midbrain sections using 81A p-aSyn (p-S129) antibody were acquired essentially as described.^16^ Five micrometer formalin-fixed paraffin embedded sections were kindly provided by Dr Ian R. Mackenzie (co-author) after the study approval by the University of British Columbia Ethics committee (Supplementary Table S2). We have also reported ancillary pathology (in the midbrain and hippocampus) of these and additional PD cases in a previous study.^16^ IHC on brain sections was performed after deparaffinization, melanin bleaching (see below) and antigen retrieval. Tissue sections were incubated with antibody against p-aSyn (S129; 1:2000) and detection was performed using the alkaline phosphatise conjugated streptavidin-biotin ABC kit (Vector Labs, #AK-5000). For destaining/bleaching of neuromelanin in substantia nigra, a slightly modified IHC protocol was used.^16^^,^^25^ Briefly, sections mounted on slides were incubated in a 60°C degrees oven for 30 min and then were transferred into ambient distilled water. Then, the slides were placed in 0.25% potassium permanganate solution for 5 min. Subsequently, the slides were rinsed with distilled water. This was followed by incubation in 5% oxalic acid until section became clear. A final rinse in distilled water was performed before proceeding with the routine IHC staining as described above. Sections were counterstained with haematoxylin (Vector Labs, #H-3401). High resolution panoramic images of tissue sections for IHC analyses were acquired using a Leica Aperio digital slide scanner.

### Nomenclature

We have employed the disease nomenclature according to Escourolle & Poirier, Manual of Basic Neuropathology, Fifth Edition^26^ and Stedman’s Medical Acronyms, Fifth Edition.^27^

### Statistical analysis

The data were statistically analysed in Graphpad Prism software (version 9), and the final graphs were prepared in Microsoft Excel. Statistical significance was calculated by the analysis of variance (ANOVA), as indicated in the figure legends.

### Data availability

All of the data generated and analysed during this study are included in the main manuscript and the associated supplementary files.

## Results

Bilateral intramuscular (IM) inoculation of mouse PFF aSyn into hindlimb (biceps femoris) in homozygous M83^+/+^ mice results in a moribund phenotype, which is characterised by footdrop and paralysis (median onset, day 53 post-injection).^13^ The M83 (heterozygous and homozygous) mice also exhibit progressive hindlimb clasping following IM inoculation with PFF aSyn, which we have observed to appear (in mild degree) after 3 weeks post-injection, is clearly established around day 45–50 and increases in severity over the next 2–3 weeks.^15^^,^^16^ This deficit in sensorimotor coordination is a recognized feature in the rodent models of neurodegeneration including motor neurone disease, cerebellar ataxia and chemical parkinsonism.^28–30^ Briefly, when symptomatic rodents are picked by the tail and slowly descended to a surface, they adopt a flexed posture and retract the hindlimbs towards the abdomen (instead of the normal response that consists of extending the limbs in anticipation of the contact^28^).

We wanted to investigate: (i) the extent of CNS aSyn pathology prior to the emergence of these sensorimotor deficits (hindlimb clasping and foot drop),^13^^,^^16^ i.e. prodromal phase, and (ii) whether the early topographical distribution of intracerebral aSyn pathology shows a propensity for distinct neuronal populations, and subsequent propagation into neuronatomically connected additional brain regions,^23^ that coincide with the emergence of senorimotor deficits. To study these features of disease progression, we injected mouse PFF aSyn bilaterally into hindlimb biceps femoris of M83^+/+^ mice (see Materials and methods), and characterized pathological aSyn accumulation in the neuraxis by immunohiostochemical (IHC) detection of phosphorylated aSyn (p-aSyn, S129), which is a robust marker for Lewy related aSyn pathology.^1^^,^^2^^,^^10^ The largest sensorimotor innervation of the hindlimb is through the sciatic nerve, which originates from lumbar spinal segments 3 and 4 (L3–4) in mice. This mixed nerve consists of the axonal processes of Golgi type I spinal α–motor neurons innervating skeletal musculature, processes of somatosensory neurons whose cell bodies reside in the spinal dorsal root ganglia (DRGs), and unmyelinated fibers of the autonomic nervous system.^23^ Therefore, we examined aSyn pathology (p-aSyn, S129) in the lumbar spinal cord and L4 DRG at the following stages: (i) fourteen days post PFF injection—dpi 14 (behavior: no clasping, freely moving); (ii) dpi 21 (behavior: mild clasping, freely moving); and (iii) dpi 45 (behavior: mild clasping, non-paralyzed).

### Incipient α-Syn pathology predominantly affects pools of spinal premotor and motor neurons following hindlimb IM inoculation with PFF aSyn

**Figure 1:**
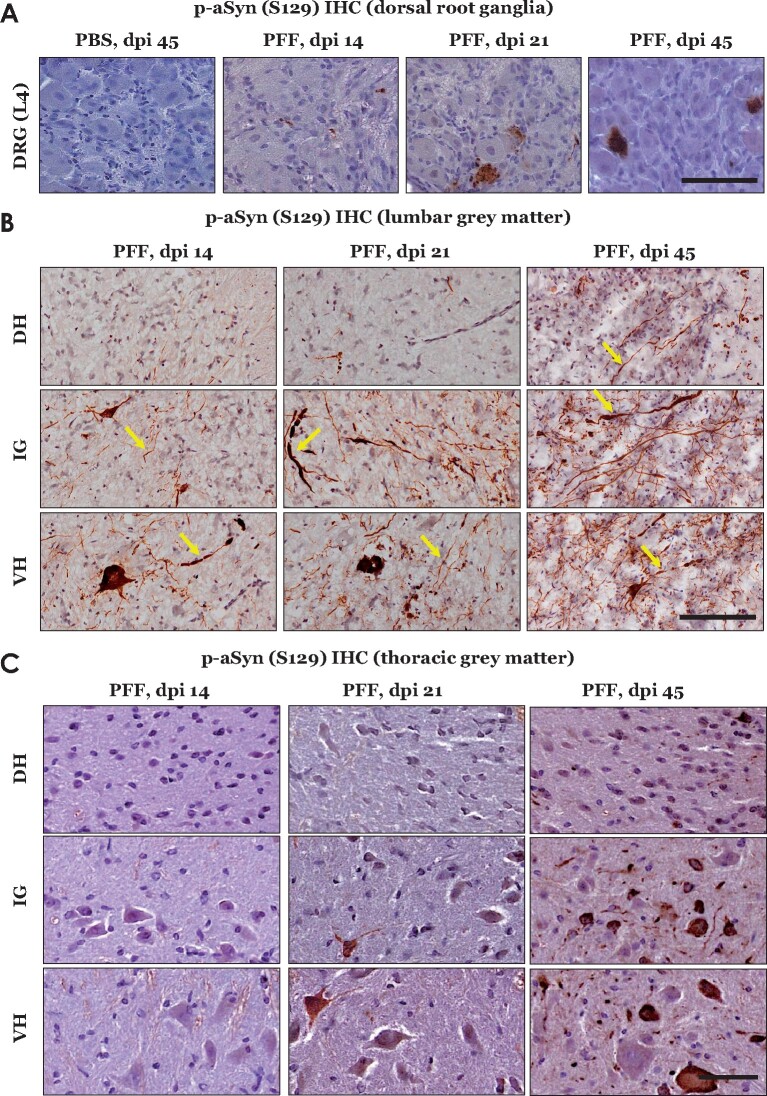
**Immunostaining of phospho-alpha synuclein (p-aSyn, S129) in dorsal root ganglia (DRG, L4) and spinal grey matter of M83^+\+^ mice.** (**A**) Representative images showing p-aSyn (S129) IHC in L4 DRG: PBS injected- dpi 45 and PFF aSyn injected- dpi 14, dpi 21 and dpi 45 (PBS, phosphate buffered saline; PFF, pre-formed alpha synuclein- aSyn fibrils; dpi, days post-injection; 40× magnified views, scale bar = 50 µm). (**B**) Representative images showing p-aSyn (S129) IHC in lumbar grey matter of PFF aSyn injected cohorts (dpi, days post-injection; DH, dorsal horn; IG, intermediate grey; VH, ventral horn; 40× magnified views, scale bar = 50 µm). Yellow arrows point to select instances of neuritic aSyn pathology. Also see Supplementary Fig. 1A, for representative images from PBS injected cohort- dpi 45. (**C**) Representative images showing p-aSyn (S129) IHC in thoracic grey matter of PFF aSyn injected cohorts (dpi, days post-injection; DH, dorsal horn; IG, intermediate grey; VH, ventral horn; 40× magnified views, scale bar = 50 µm). Primary antibody in **A–C**: p-aSyn (S129)- 11A5.

**Figure 2:**
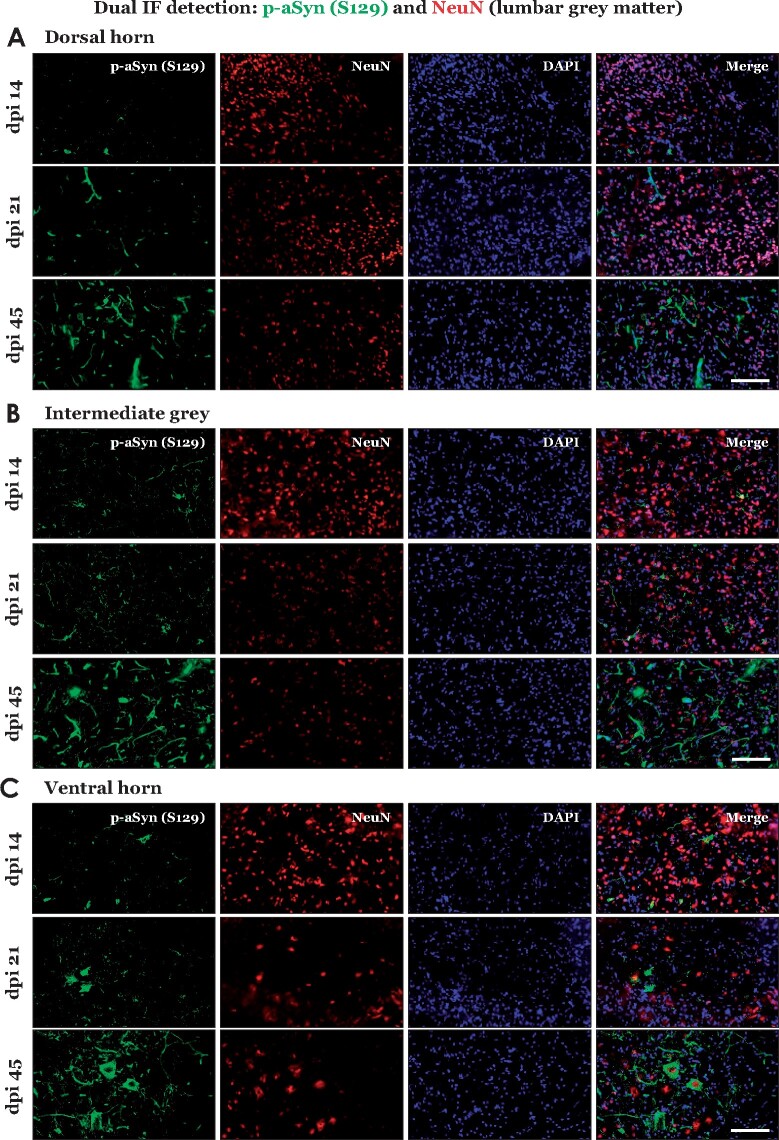
**Immunofluorescence detection of phospho-alpha synuclein (p-aSyn, S129) in the lumbar spinal grey matter of PFF aSyn injected M83^+\+^ mice.** (**A–C**) Representative images showing dual immunofluorescence detection of p-aSyn (S129, in green) and neuronal nuclei marker (NeuN, in red) in lumbar grey matter of PFF aSyn injected cohort: dorsal horn (**A**), intermediate grey (**B**) and ventral horn (**C**). (dpi, days post-injection; 40× magnified views, scale bar = 50 µm). Also see Supplementary Fig. 1B, for representative images from the PBS injected cohort- dpi 45. Primary antibodies in **A–C**: p-aSyn (S129)- 11A5 and NeuN- A60.

Our IHC analyses of L4 DRG from PFF aSyn injected cohorts indicated sparse p-aSyn S129 immunostaining at early stages (dpi 14 and dpi 21), which subsequently became pronounced at dpi 45 (Fig. 1A). In contrast, substantial p-aSyn S129 immunostaining was detected in the lumbar spinal cord as early as 14 dpi (Fig. 1B), predominantly in the ventral horn (VH) and intermediate grey (IG), which harbour pools of premotor and motor neurons.^23^^,^^31^ Cell populations in the dorsal horn (DH)—predominantly nociceptor neurons and interneurons of the somatosensory system^23^ –did not show comparable p-aSyn S129 accumulation at these stages except a mild degree of staining in neurites (Fig. 1B, dpi 14 and dpi 21). By dpi 45, aSyn pathology—both in the cell bodies and neurites—was conspicuous in the DH, IG and VH (Fig. 1B, dpi 45; also compare Supplementary Fig. 1A—PBS injected cohort, dpi 45). To further characterize the spinal cord pathology, we assessed p-aSyn S129 immunostaining in the thoracic spinal cord, which does not directly innervate the hindlimb injection site (hence, spreading in this region would indicate the involvement of central projections, and not thorough the sciatic nerve peripheral afferents). We observed a visible lack of aSyn pathology in the thoracic cord at dpi 14, when significant VH and IG pathology was present in the lumbar cord (Fig. 1C, compare with Fig. 1B). However, progressive accumulation of p-aSyn (S129) was detected in the thoracic cord at dpi 21 and dpi 45, predominantly affecting the VH and IG compartments (Fig. 1C). Double IF detection with neuronal nuclei marker (NeuN) in the lumbar cord confirmed that p-aSyn S129 immunostaining was primarily detected in neuronal populations of spinal grey matter (Fig. 2A–C; also see Supplementary Fig. 1B—PBS, dpi 45), and was present in large NeuN immunopositive, α-motor neurons of the VH.^32^ Furthermore, neuronal p-aSyn S129 immunostaining also exhibited co-detection with the p62 protein (Sequestosome 1, a marker of intracellular protein aggregates and autophagy) at all stages (Supplementary Fig. 1C); albeit, a few p-aSyn S129 immunopositive but p62 immunonegative cells were also seen at dpi 21 (Supplementary Fig. 1C, dpi 21—indicated by the white arrow). Collectively, these observations indicate the preferential affection of the spinal premotor and motor projections during the course of early stages (following intramuscular PFF aSyn inoculation), and suggest secondary involvement of the sensory neuronal populations in DH as the pathology became widespread.

### Peripherally induced prodromal α-Syn pathology affects brainstem reticular nuclei and subsequently propagates into regions controlling movement and posture

In addition to the descending projections of the upper motor neurons, the spinal somatomotor system receives an extensive array of descending projections, predominantly originating in the brainstem nuclei: medullary and pontine reticular nuclei, locus coeruleus (LC), red nucleus (RN), vestibular nuclei (VN) and (indirectly) cerebellum.^5^^,^^23^^,^^33^ These extrapyramidal inputs provide a gain setting mechanism for the motor system, and modulate muscle tone, posture and balance.^5^^,^^23^^,^^33^

**Figure 3:**
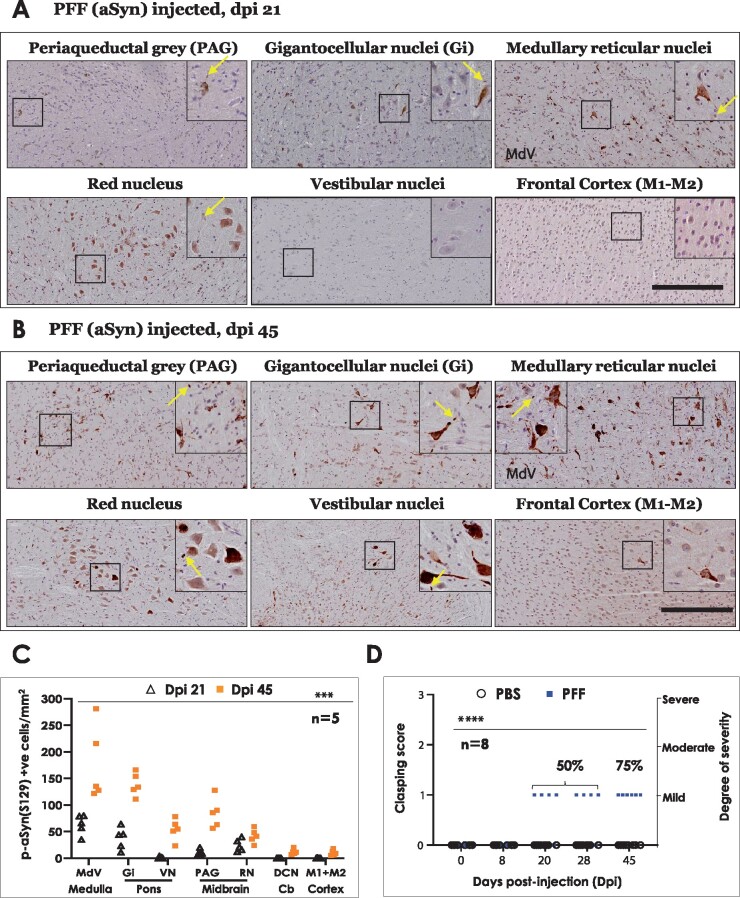
**Immunostaining of phospho-alpha synuclein (S129) in brain regions of PFF aSyn injected M83^+\+^ mice and assessment of hindlimb clasping behaviour.** (**A–B**) Representative images showing p-aSyn (S129) IHC in indicated brain regions at dpi 21 (**A**) and dpi 45 (**B**) (dpi, days post-injection; 10× magnified views, scale bar = 200 µm). Yellow arrows in the insets point to select instances of p-aSyn (S129) positive oligodendroglial cells in the vicinity of neurons. Also see Supplementary Fig. 2A–B (representative images from PBS injected- dpi 45 and PFF aSyn injected- dpi 14 cohorts) and Supplementary Fig. 3A (5× low magnification images annotated with neuroanatomical landmarks, PFF aSyn injected- dpi 45). Primary antibody in **A–B**: p-aSyn (S129)- 11A5. (**C**) Semi-quantitative analyses of p-aSyn (S129) immunopositive cells in the indicated brain regions at dpi 21 and dpi 45 in PFF injected M83^+/+^ mice. Individual data point represents p-aSyn (S129) cell counts/mm^2^/region in each animal of the respective cohort, with dpi 21 being depicted as blank triangles and dpi 45 as the orange squares. (MdV, medullary reticular nuclei; Gi, pontine gigantocellualr nuclei; VN, pontine vestibular nuclei; PAG, periaqueductal grey matter; RN, red nucleus, DCN- crb, deep cerebellar nuclei; M1+M2, primary and secondary motor cortex; One-way ANOVA, Kruskal–Wallis test- p.0002, *n* = 5/group; dpi, days post-injection). (**D**) Hindlimb clasping assessment in PBS and PFF aSyn injected cohorts, with individual data points representing clasping score of each animal in the respective cohort. Notice that PBS injected mice do not exhibit hindlimb clasping (blank circles, score = 0). The PFF injected mice start show mild clasping around dpi 21–28 and later (blue squares; 4 animals at dpi 21–28 and 6 animals at dpi 45). Two-way ANOVA (alpha 0.05- *P* < 0.0001; *n* = 8/group).

Therefore, we assessed spatiotemporal emergence and progression of aSyn pathology (p-aSyn S129) in these brain regions. Our IHC analyses showed lack of appreciable p-aSyn S129 immunostaining in brain, either developing spontaneously in M83^+/+^ mice (Supplementary Fig. 2A, PBS dpi 45), or at dpi 14 following PFF aSyn IM injection (Supplementary Fig. 2B). Subsequently (dpi 21), we detected intracerebral p-aSyn S129 immunostaining, with particular abundance in the reticular nuclei of hindbrain including medullary reticular nuclei (Fig. 3A, MdV) and pontine gigantocellular nuclei (Fig. 3A, Gi), as well as in the periaqueductal grey (PAG) and RN in the midbrain region (Fig. 3A). Among other regions, there was clear lack of p-aSyn S129 immunostaining at this stage, including VN or motor cortex regions (Fig. 3A). By dpi 45, additional neuronal populations were found to be p-aSyn S129 immunopositive, predominantly in the regions affected at dpi 21 mentioned above (Fig. 3B–C). Intriguingly, we also noticed scant p-aSyn S129 immunostaining in the deep cerebellar nuclei (DCN; lateral and interposed), which do not directly synapse with the spinal motor compartment^23^ (Supplementary Fig. 2C, dpi 45). Additional aSyn neuronal pathology was found in the dorsomedial hypothalamus (DM) and nucleus of the oculomotor nerve (Supplementary Fig. 3A: 5× views annotated with neuroanatomical landmarks, and 40× high magnification views). In addition, some regions either showed: (i) comparably weaker p-aSyn S129 immunostaining (Supplementary Fig. 2C, midbrain tegmentum containing substantia nigra and ventral tegmental area—parts of nigrostriatal circuitry; and Supplementary Fig. 3A, ventralposterior thalamus—principal relay nuclei of the ascending somatatosensory projections), or (ii) it was almost non-existent (Supplementary Fig. 2C, corpus striatum). Remarkably, the neuronal aSyn pathology in the affected regions was also associated with p-aSyn S129 immunopositive neurites and oligodendrocytes (myelin producing cells in the CNS) in the vicinity (Fig. 2A and B, yellow arrows in the insets).

Since reactive astrogliosis, astroglial accumulation of p-aSyn S129 in the spinal grey matter and neuroinflammation has been reported as a pathological feature in the late (terminal) stage M83 mice,^13^^,^^34^ we also probed these tissue reactions in the CNS (dpi 14, dpi 21 and dpi 45). Double IF detection of p-aSyn S129 and the astroglial marker glial fibrillary acidic protein (GFAP) revealed comparatively mild astrogliosis, without remarkable detection of aSyn pathology in the astrocytes (Supplementary Fig. 4A–C). We also examined select brain regions harbouring significant p-aSyn (S129) immunopositive neurons at dpi 45 (Fig. 3B and C; PAG, Gi and VN), and did not detect extensive astrogliosis or phagocytes infiltration (Supplementary Fig. 5A and B). These observations suggest that neuroinflammatory tissue reactions in the CNS emerge as a late feature in this model of synucleionopathy. These results are also in line with findings in heterozygous M83^+/−^ mice in which reactive gliosis and phagocyte infiltration appears around 10–12 weeks post-injections, despite the presence of significant aSyn pathology as early as 4 weeks post-PFF aSyn injection.^17^^,^^34^

Lastly, in order to assess the effects of these early stages of aSyn pathology on a known behavioural deficit in this model,^15^^,^^16^ we also performed hindlimb clasping over the course of the studies. We found that the PFF aSyn cohorts exhibited a mild clasping phenotype which was discernible at dpi 21 (50% animals) and dpi 45 (75% animals) (Fig. 3D; also see Methods). These data demonstrate that the early phase of intracerebral pathology is consistent with the retrograde neuroinvasion of aSyn affecting distinct projections of the somatomotor system (Fig. 3A, dpi 21), and not the ascending projections of the spinal somatosensory neurons (anterograde route). However, the later phase(s) of intracerebral propagation into VN, DCN or DM (Fig. 3B, dpi 45), suggest additional routes, with significant involvement of LC, PAG and hindbrain reticular nuclei^23^^,^^33^^,^^35^ (elaborated in Discussion).

### PFF aSyn induced early α-Syn pathology in CNS of M83^+/+^ mice leads to ancillary oligodendroglial p-aSyn S129 accumulation in the white matter tracts

**Figure 4:**
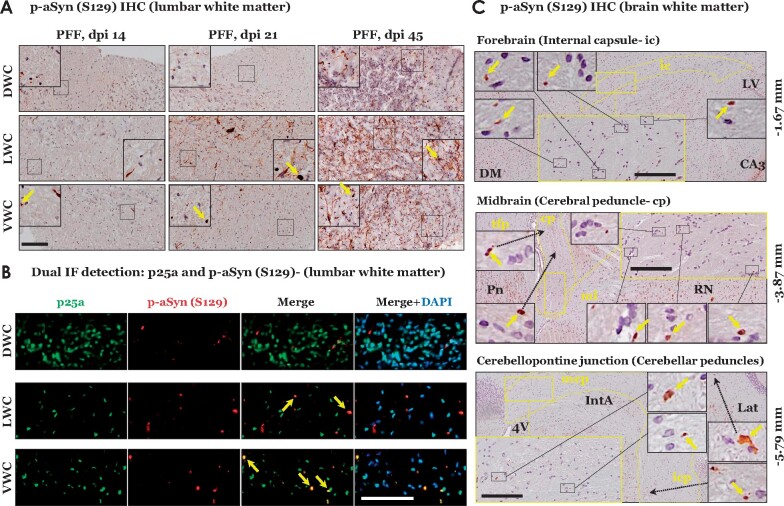
**Immunostaining of phospho-alpha synuclein (S129) within white matter regions of PFF aSyn injected M83^+\+^ mice.** (**A**) Representative images showing p-aSyn (S129) IHC in lumbar white matter columns of PFF aSyn injected cohorts- dpi 14, dpi 21 and dpi 45 (dpi, days post-injection; DWC, dorsal white column; LWC, lateral white column; VWC, ventral white column; 20× magnified views, scale bar = 50 µm). Yellow arrows in the insets point to select instances of p-aSyn (S129) positive oligodendroglial cells. Primary antibody in **A**: p-aSyn (S129)- 11A5. (**B**) Representative images showing dual immunofluorescence detection of oligodendroglial marker (p25a, in green) and p-aSyn (S129, in red) in lumbar white matter of PFF aSyn injected cohort- dpi 45 (dpi, days post-injection; DWC, dorsal white column; LWC, lateral white column; VWC, ventral white column; 40× magnified views, scale bar = 50 µm). Merge images with DAPI (nuclear stain, in blue) are also shown. Primary antibodies in 4B: p-aSyn (S129)- 11A5 and p25a. (**C**) Representative images showing p-aSyn (S129) IHC in indicated white matter tracts in brains of PFF aSyn injected cohort- dpi 45 (5× low magnification views, and areas highlighted within the yellow rectangles are shown as 20× magnified views, scale bar = 100 µm). Yellow arrows in the insets point to select instances of p-aSyn (S129) positive oligodendroglial cells in the white matter tracts. Bregma co-ordinates (in mm) are derived from Paxinos and Franklin’s (Fourth edition 2013, Elsevier) with the following topographical landmarks: (Forebrain) ic, internal capsule; LV, lateral ventricle; CA3, cornu ammonis area 3 of hippocampal formation; DM, dorsomedial hypothalamus; (Midbrain) cp, cerebral penduncle; tfp, transverse pontine fibres; ml, medial leminiscus; RN, red nucleus; Pn, pontine nuclei; (Cerebellopontine junction) mcp, middle cerebellar penduncle; icp, inferior cerebellar peduncle; 4V, fourth ventricle; IntA, interposed cerebellar nuclei; Lat, lateral cerebellar nuclei). Primary antibody in **C**: p-aSyn (S129)- 11A5.

Our observations concerning the substantial affection of oligodendroglial cells in the vicinity of neurons harbouring early neuronal aSyn pathology prompted us to investigate whether the oligodendroglial lesions are a local feature in these brain regions (Fig. 3A and B, yellow arrows in insets), or is a more widespread phenomenon in the CNS of PFF aSyn (IM injected) M83^+/+^ mice. Oligodendroglial pathological aSyn lesions are a defining feature in MSA, another neurodegenerative syncleinopathy affecting the CNS.^19^^,^^36^ Moreover, previous studies (using direct inoculation of mouse PFF aSyn in the sciatic nerve of heterozygous M83^+/−^ mice) have shown that significant oligodendroglial aSyn pathology in the spinal white matter is a characteristic finding at the terminal stage (3.9 ± 0.1 months).^18^ Therefore, we assessed p-aSyn S129 immunostaining in the oligodendroglia within the white matter tracts at the level of lumbar spinal cord, and within the large tracts of the somatomotor system in the brain. We detected sparse oligodendroglial p-aSyn S129 accumulation, notably in the lateral and ventral white matter columns of lumbar cord at dpi 14 and dpi 21 (Fig. 4A). At dpi 45, p-aSyn S129 immunopositive oligodendrocytes were found in all white matter regions in the lumbar cord—as shown by IHC (Fig. 4A) or double IF labelling with oligodendroglial marker p25α (Tubulin Polymerization Promoting Protein) (Fig. 4B)—with predominant involvement of lateral and ventral white matter spinal columns. Furthermore, pathological aSyn (p-aSyn, S129) lesions were also detected in the large white matter tracts of the brain, including the internal capsule in the forebrain, mesencephalic cerebral peduncles and (sparsely) in the cerebellar peduncles in the hindbrain (Fig. 4C, dpi 45). In addition to supporting the notion that pathological aSyn propagation occurs along neuroanatomical tracts,^11^ these observations demonstrate that the white matter aSyn pathology in this model is a feature during the early stages of the disease progression, and is more widespread than previously anticipated.^13^^,^^18^

**Figure 5:**
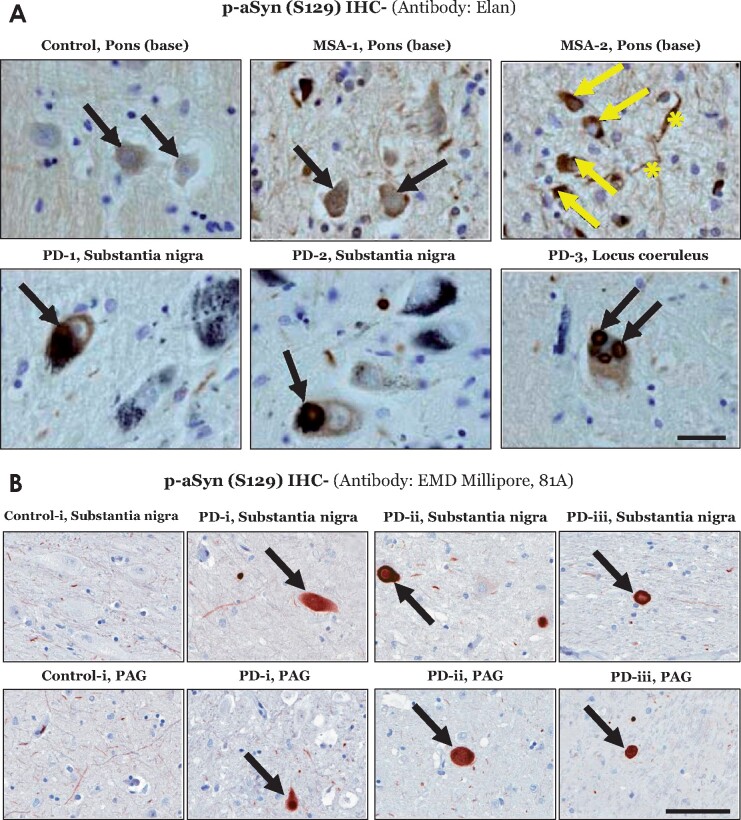
**Immunostaining of phospho-alpha synuclein (p-aSyn, S129) in post-mortem human brain sections.** (**A**) Representative images showing p-aSyn (S129) immunostaining by the Elan antibody in a control (base of pons- basis pontis), two multiple system atrophy cases (MSA, #1–2, Supplementary Table S1; base of pons) and three Parkinson disease cases (#1–3, Supplementary Table S1; substantia nigra and locus coeruleus). The black arrows indicate neuronal p-aSyn (S129) inclusions in both Parkinson disease and MSA cases. In MSA-2, glial p-aSyn (S129) inclusions are indicated by the yellow arrows, while the fibre tract staining is indicated by the yellow asterisks (scale bar = 20 µm). (**B**) Representative images showing p-aSyn (S129) immunostaining by the EMD Millipore antibody 81A in the midbrain substantia nigra and periaqueductal grey-PAG of a control (#i, Supplementary Table S2) and three Parkinson disease cases (#i–iii, Supplementary Table S2). The black arrows indicate Lewy body pathology in Parkinson disease (scale bar = 100 µm). Also see Supplementary Fig. 6.

Overall, p-aSyn (S129) immunostaining in the affected CNS regions of M83^+/+^ mice microscopically had a homogeneous appearance, and was diffusely localized in a predominantly cytosolic pattern (Figs. 1–4, Supplementary Fig. 3). However, Lewy related aSyn neuronal pathology in human PD studies, using aSyn and/or p-aSyn (S129) IHC, exhibits a spectrum of morphological and ultrastructural features, including compact organization as cytosolic pale bodies and LBs with a halo.^1^^,^^2^^,^^37^ In MSA affected brain, pathological aSyn accumulation- in the form of neuronal and oligodendroglial cytoplasmic inclusions (GCIs)- is found in a widespread distribution in brainstem and cortex, with co-incidental aSyn lesions in the related white matter tracts.^38^^,^^39^ In this context, it has been suggested that the diffuse cytosolic immunostaining pattern detected with some p-aSyn (S129) antibodies could be an indicator of distinct Lewy related aSyn lesions in synucleionopathies [reviewed in Refs.^1^^,^^2^^,^^39^]. We have also observed discrete instances of diffuse cytoplasmic localization of p-aSyn (S129) in MSA and PD post-mortem brain sections by p-aSyn (S129) IHC. For instance, using the Elan p-aSyn (S129) antibody (mouse mAb) IHC of MSA affected tissue (*basis pontis*), we observed homogenous, predominantly cytoplasmic, immunostaining in neuronal soma, neurites and oligodendroglia (Fig. 5A). Similarly, in PD brain sections, we observed instances of this gross microscopic immunostaining pattern of p-aSyn (S129) antibodies [Fig. 5A, PD midbrain and pons, Elan p-aSyn (S129) antibody; Fig. 5B and Supplementary Fig. 6B, PD midbrain regions, 81A antibody]; albeit, inclusions with additional morphological patterns characteristic of LB pathology were also found (Supplementary Fig. 6B, PD substantia nigra). Although the gross appearance of p-aSyn (S129) lesions in the CNS of M83^+/+^ model reflects some degree of resemblance to Lewy related aSyn pathology in human tissue specimen, these data should be interpreted with caution and require further systematic analyses to establish the relevance of this feature to synucleinopathies (see Limitations of the model, in Discussion).

## Discussion

The clinical presentation of PD is defined by somatomotor dysfunction in the nigrostriatal system, hence understanding the mechanisms underlying the loss of dopaminergic projection neurons in the SN have been major topic of interest over the last two decades.^3^^,^^4^ Nevertheless, the neuropathological staging proposed by Braak emphasizes the role of extranigral aSyn pathology in PD, in particular the early affection of brainstem nuclei in the aetiology of autonomic disturbances, sleep disorder and somatomotor incoordination.^5^^,^^8^^,^^33^ It is hypothesized that pathological aSyn originates in periphery (e.g. gut, olfactory mucosa), propagates in a ‘prion-like’ fashion from these early affected regions, and progressively involves brain regions following their neural connectivity.^8^^,^^11^ Therefore, efforts to understand the prodromal phase of PD has gained significant interest, and guided the design of experimental models for studying the extranigral aSyn pathology in the context of disease propagation and pathogenesis.^11^

### Spatiotemporal features of early aSyn templating in CNS following intramuscular route

**Figure 6:**
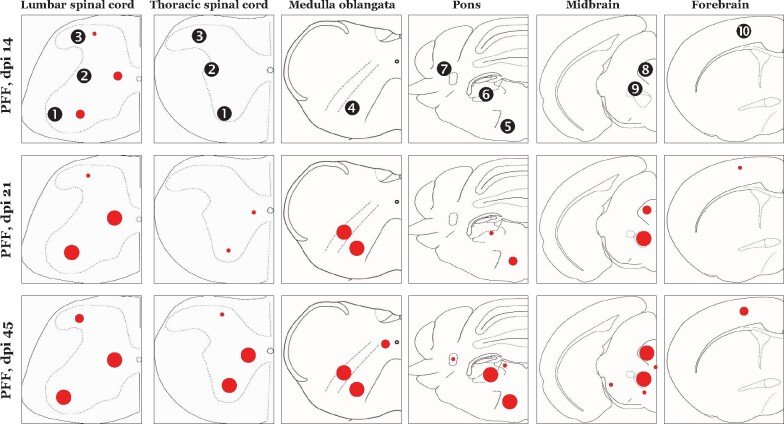
**Composite schematic depiction of the early stage phospho-alpha synuclein (p-aSyn, S129) immunodetection in the CNS of PFF injected M83^+/+^ mice.** Average number of p-aSyn (S129) immunopositive cells in the regions is indicated by the red circles (small circle, 1–10 cells/mm^2^; medium circle, 10–30 cells/mm^2^; large circle, >30 positive cells/mm^2^). Regions shown: (Spinal cord) ventral horn, intermediate grey, dorsal horn; (Medulla oblangata) MdV, medullary reticular nuclei; (Pons) gigantocellualr nuclei, vestibular nuclei, cerebellar nuclei; (Midbrain) periaqueductal grey, ed nucleus; (Forebrain) motor cortex [dpi, days post-intramuscular injection with preformed fibrililar (PFF) aSyn; Structures are not drawn to scale]. Also see Figs. 1–3 and Supplementary Fig. 3.

We have attempted to delineate the emergence and propagation of early aSyn (p-aSyn, S129) pathology in the nervous system of a mouse model of synucleinopathies (M83^+/+^ line). Notably, we studied these features in non-paralyzed (PFF aSyn injected) M83^+/+^ animals, and have provided a spatiotemporal paradigm highlighting the progression of aSyn pathology of peripheral origin via the intramuscular route (Fig. 6 and Graphical abstract). Our data show that characteristic aSyn pathological lesions in extranigral regions of brain, predominantly within the reticular nuclei of the brainstem (Fig. 3; Supplementary Fig. 3), were present long before the emergence of debilitating defects in sensorimotor functions. In other words, the mice were freely moving and exhibited a mild degree of hindlimb clasping (Fig. 3D), a behaviour indicating dysfunction in sensorimotor coordination and posture maintenance.^28^^,^^30^ This phenotype is distinct than the terminal stage, which is characterized by a moribund phenotype including hindlimb paralysis, hunched posture and severe degree of hindlimb clasping.^13^^,^^15^^,^^16^ However, we cannot rule out subtle neuronal dysfunction, and given the significant white matter involvement, defects in nerve conduction that could be assessed by sensitive behavioural assays (i.e. electrophysiology, electromyography and tests of proprioceptive function^40^^,^^41^) In this regard, we have also reported that IM inoculation (in gastrocnemius muscle) of PFF aSyn in heterozygous M83^+/−^ mice is associated with degeneration of small and medium sized myelinated fibres in the sciatic nerve, reduced nerve conduction velocity and abnormal pain perception (mechanical allodynia), as early as 45 days post-injection.^15^ Similarly, IM injection of PFF aSyn in heterozygous M83^+/−^ mice has also been shown to affect brain microstructural and resting-state functional activity as early as four weeks post-injection, significantly preceding the detection of intracerebral α-syn pathology (detected around 12 weeks post-injection) or gross locomotor defects.^17^

In addition to providing the early spatiotemporal context to the terminal stage aSyn pathology in CNS following PFF aSyn inoculation in M83^+/+^ mice,^13^^,^^16^ our observations corroborate the notion concerning the propensity of aSyn seeds in affecting distinct neuronal populations and/or propagation through distinct neuroanatomical tracts.^11^^,^^18^ The gross microscope appearance of early stage neuronal p-aSyn (S129) exhibited a homogenous cytosolic pattern of staining (Fig. 1, Fig. 3 and Supplementary Fig. 3). It is tantalizing to consider if these intraneuronal aSyn accumulations also contain oligomeric conformations, in addition to the fibrillar deposits characteristic of Lewy related aSyn pathology. However, to qualify them as *bona fide* fibrillar and/or oligomeric intracellular deposits requires an array of analyses including immunostaining for multiple markers/LB components, assessment of biochemical composition, and advanced ultrastructural characterization.^1^^,^^2^^,^^39^ In a limited number of PD brain specimen, we observed LB inclusions which resembled this morphological pattern, along with additional morphologies characteristic of LBs including peripherally localized cytoplasmic inclusions (Fig. 5A and B, Supplementary Fig. 6B). A recent opinion alludes that distinct LB morphologies (in conjunction with biochemical and ultrastructural analyses) could be used to guide a staging system.^2^ However, these observations (Fig. 5A–B and Supplementary Fig. 6B) require due caution in interpretation, until further analytical and experimental evidence becomes available.

Our results advance previous findings reported in the PFF M83 (IM inoculation) model with a few notable differences: (i) direct sciatic nerve inoculation (in contrast to the intramuscular route) of PFF aSyn also resulted in predominant affection of the somatomotor spinal system and reticular nuclei, however, significant pathological aSyn accumulation in brain was found after 2 months post-injection^18^ (most likely due to the gene dosage effect in heterozygous animals); (ii) we found that aSyn pathology in white matter oligodendroglial cells not only developed earlier (14–21 dpi), but also was progressive in nature and more widespread in CNS (Figs. 3 and 4) than observed previously^13^^,^^18^; and (iii) we did not observe robust inflammatory gliosis or glial accumulation of pathological aSyn at these early stages (Supplementary Figs. 4 and 5), as reported in the terminal stage studies.^13^^,^^34^ We consider these tissue reactions to be among the late events of neuroinflammation, and this is supported by studies showing the development of astrogliosis 3–4 months post-injection (involving PFF aSyn IM inoculation in gastrocnemius of heterozygous M83^+/− 17,34^ or in a related PFF mouse model: aSyn A53T-GFP).^42^

### Implications for novel mechanistic paradigms in PD and related synucleinopathies

Based on these data, the mechanistic aspects of aSyn pathology initiation, and subsequent stages of propagation in CNS can be grossly studied as distinct phases, in relation to the sensorimotor performance in PFF injected M83^+/+^ model (Graphical abstract). The neuroanatomical connectivity of early affected neuronal populations in spinal somatomotor system and brainstem reticular nuclei is extensive, and the regulation of their activity and functions are beyond the scope of this discussion [The readers are referred to study these details elsewhere^31^^,^^35^^,^^43^^,^^44^]. Nevertheless, we consider the following points noteworthy for consideration in future studies:

**The spreading of pathological aSyn ‘seeds’ from muscle to the spinal motor components:** It is plausible that the propagation of pathological aSyn occurs in a retrograde manner through the ventral root nerve efferents, and subsequently affects the cell bodies of motor neurons in the spinal ventral horn. However, the concomitant appearance of early aSyn pathology (Fig. 1) in spinal grey matter regions harbouring premotor and proprioceptive neurons,^23^^,^^31^ and some neurons within L4 DRGs suggest additional route(s). In this context, we consider that the muscle spindles (containing intrafusal, non-contractile, muscle fibres) also represent a major and/or ancillary route. These encapsulated muscle stretch receptors establish monosynaptic contacts on spinal neuronal populations via Ia sensory afferents, and mediate the spinal stretch reflex and unconscious proprioception.^23^^,^^45^ In this regard, altered morphology and/or functional connectivity of muscle spindles have been implicated in proprioceptive defects in PD and other neurodegenerative diseases.^45^^,^^46^ The role of Ia afferents in aSyn propagation could be studied by carrying out selective transection of the ventral or dorsal roots at the level of spinal DRGs.

**New experimental models of extranigral neuronal aSyn pathology in PD:** Within brainstem, our data indicate the early involvement of the neuronal populations contributing to the nuclei of the reticular formation, i.e. MdV, pontine gigantocellular (Gi) nuclei and midbrain PAG (Fig. 3A, dpi 21). Based on the widespread connectivity of these regions,^35^^,^^43^ it is tempting to investigate if selective induction of aSyn pathology in (one or more of) these regions recapitulates the pattern of pathological aSyn propagation, and more importantly, results in distinct behavioural deficits relevant to PD, i.e. posture and gait maintenance. In this regard, neuropathological studies in PD^8^^,^^33^ and DLB show that aSyn neurodegenerative pathology in Gi, LC and PAG is not a late event.^5^^,^^36^ Pathological involvement of cerebellum or vestibular nuclei (connected via extensive reciprocal projections^23^) in PD has been reported in a limited number of studies;^36^^,^^47^ however, these features require further evaluation.

**‘Periphery-first’ and ‘brain-first’ hypotheses in the aetiology of PD:** These hypotheses have been proposed to reconcile the aetiology of PD subtypes which are not explained by the ‘retrograde’ propagation of pathological aSyn from periphery into CNS. According to this notion, pathological aSyn in some PD subtypes originates in brain and ‘anterogradely’ propagates into the peripheral nerve terminals (e.g. in the gut).^48^ In addition, mathematical models suggest that the initial phase of transynaptic transfer of pathological aSyn occurs in a retrograde manner; however, late-stage events fit the ‘anterograde’ model.^49^ With reasonable caution, there are few observations in our data that could be further exploited to elucidate these mechanisms: (i) the delayed emergence of aSyn pathology in the DH, both in lumbar (Fig. 1B) and thoracic segments (Fig. 1C): While the lumbar DH pathology can be easily explained by direct spread from the lumbar DRGs (Fig. 1A), the involvement of DH in thoracic cord is puzzling. As the DH neuronal populations (nociceptor, autonomic and propiospinal neurons) receive substantial descending projection of a modulatory nature, such as from the reticular nuclei,^33^^,^^43^ it will be highly informative if the selective induction of aSyn pathology in these higher brain regions would ‘anterogradely’ affect spinal neurons through their descending projections. In this context, Braak and colleagues have reported that in a subset of PD cases, pathological aSyn lesions are detected in the lamina I and autonomic nuclei in the thoracic cord, although the authors implicated the enteric nervous system as the site of origin^50^ and (ii) White matter aSyn pathology in the CNS: Our data (Fig. 4) highlight the ancillary affection of the white matter tracts, and significant oligodendroglial aSyn pathology in the M83^+/+^ model. The white matter lesions were remarkably pronounced within the lateral and ventral white matter columns in the spinal cord (in contrast to the dorsal column—ascending somatosensory tracts), and also within the large descending motor tracts in the brain. At this stage, we do not know if p-aSyn S129 containing oligodendrocytes (Fig. 4) are associated with local axonal projections of the affected neurons (Fig. 3A and B), or ensheath the descending projections of intracerebral origin.

### Limitations of the model

While the inoculation of exogenous pathological aSyn in the M83 and similar models is useful in studying the effects of aggressive aSyn pathology induction in CNS, and in investigating the sensorimotor defects due to extra-nigral p-aSyn (S129) accumulation,^15^^,^^17^ certain limitations potentially affecting the choice of experimental paradigm are worth mentioning: (i) the distribution of CNS aSyn pathology and onset of the motor phenotype is affected by several experimental factors including the dosage and nature of PFF aSyn inoculum (i.e. human or mouse), and the site of inoculation (e.g. brain parenchyma, olfactory bulb, peripheral nerves or muscle)^51^^,^^52^; (ii) the genetic background (e.g. the effects of neuronal aSyn expression due to *Prnp* promoter) may also affect the observed distribution of pathological lesions, and relative lack of neurodegeneration in nigrostriatal circuitry^11^^,^^53^; iii) the intraneuronal aSyn pathology (Figs. 1–3, and Supplementary Fig. 3) does not recapitulate the morphological spectrum of LB related lesions observed in human synucleinopathies (Fig. 5A–B and Supplementary Fig. 6B), and requires robust systematic analyses (i.e, biochemical studies, IHC with several antibodies and ultrastructural profiling^1^^,^^2^) for establishing its relevance to disease pathogenesis; and iv) progression to severe motor disability leading to reduced survival is rapid, and occurs within 2–3 weeks after the onset of initial defects in movement co-ordination (e.g. unilateral foot drop),^13^ potentially representing a disadvantage from a therapeutical aspect.^53^

## Conclusion

In conclusion, our study contributes to critical advances in deciphering the early events during aSyn neuroinvasion in this rodent model of peripheral-to-central aSyn pathology propagation. The knowledge of the spatiotemporal features of aSyn pathology presented herein points to a potential window of therapeutic opportunity, both for evaluating the therapy mode of action in blocking peripheral-to-central propagation of aSyn pathology, as well as in early-stage disease modification.

## Supplementary material

Supplementary material is available at *Brain Communications* online.

## Supplementary Material

fcab104_Supplementary_Data

## Abbreviations

aSyn =: α-synuclein
DCN =: deep cerebellarnuclei
DH =: dorsal horn
DM =: dorsomedial hypothalamic nuclei
Dpi =: days post-injection
DRG =: dorsal root ganglia
Gi =: gigantocellular nuclei
IF =: immunofluorescence
IG =: intermediate grey matter
IHC =: immunohistochemistry
IM =: intramuscular
LB =: Lewy bodies
LC =: Locus coeruleus
LN =: Lewy neurites
MdV =: medullary reticular nuclei
MSA =: multiple system atrophy
PAG =: periaqueductal grey
PD =: Parkinson disease
PFF =: pre-formed fibrils
Prnp =: prion promoter
RN =: red nucleus
VH =: ventral horn
VN =: vestibular nuclei
