# Correction to: Prodromal neuroinvasion of pathological α-synuclein in brainstem reticular nuclei and white matter lesions in a model of α-synucleinopathy

**DOI:** 10.1093/braincomms/fcac067

**Published:** 2022-04-04

**Authors:** Nelson Ferreira, Mette Richner, Amelia van der Laan, Ida Bergholdt Jul Christiansen, Christian B. Vægter, Jens R. Nyengaard, Glenda M. Halliday, Joachim Weis, Benoit I. Giasson, Ian R. Mackenzie, Poul H. Jensen, Asad Jan

Nelson Ferreira, Mette Richner, Amelia van der Laan, Ida Bergholdt Jul Christiansen, Christian B Vægter, Jens R Nyengaard, Glenda M Halliday, Joachim Weis, Benoit I Giasson, Ian R Mackenzie, Poul H Jensen, Asad Jan, Prodromal neuroinvasion of pathological α-synuclein in brainstem reticular nuclei and white matter lesions in a model of α-synucleinopathy, *Brain Communications*, Volume 3, Issue 2, 2021, fcab104, https://doi.org/10.1093/braincomms/fcab104

In the originally published version of this manuscript, the eighth author's name was incorrect. The eighth author's name should read “Joachim Weis” instead of “Joachim Weiss”. This error has been corrected.

