# Supplementary material for: Prodromal neuroinvasion of pathological α-synuclein in brainstem reticular nuclei and white matter lesions in a model of α-synucleinopathy
Source: Brain Commun. 2021 May 14;3(2):fcab104. doi: 10.1093/braincomms/fcab104 (PMC8202146; doi:10.1093/braincomms/fcab104)
Supplement: fcab104_Supplementary_Data [file fcab104_supplementary_data.pdf]

TITLE: Prodromal neuroinvasion of pathological α-Synuclein in brainstem reticular nuclei and white matter lesions in a model of α-Synucleinopathy.

Authors: Nelson Ferreira[¶], Mette Richner[‡], Amelia van der Laan[¶], Ida Bergholdt Jul Christiansen[¶], Christian B. Vægter[‡], Jens R. Nyengaard[†], Glenda M. Halliday[#], Joachim Weiss°, Benoit I. Giasson*, Ian R. Mackenzie[‡], Poul H. Jensen[¶], Asad Jan[¶§]

Author Affiliations: [¶]Danish Research Institute of Translational Neuroscience (DANDRITE), Department of Biomedicine, Aarhus University, Ole Worms Allé 3, DK-8000 Aarhus C, Denmark; [‡]DANDRITE, Nordic-EMBL Partnership for Molecular Medicine, Aarhus University, Høegh-Guldbergs Gade 10, DK-8000 Aarhus C, Denmark; [†]Core Center for Molecular Morphology, Section for Stereology and Microscopy Department of Clinical Medicine, Aarhus University, Department of Pathology, Aarhus University Hospital, DK-8200 Aarhus N, Denmark; °Institute of Neuropathology, RWTH Aachen University Hospital, Pauwelsstrasse 30, 52074 Aachen, Germany; *Department of Neuroscience, University of Florida, Gainesville, Florida 3261, USA; [#]Brain and Mind Centre and Faculty of Medicine and Health, School of Medical Sciences, University of Sydney and Neuroscience Research Australia, Sydney 2006, Australia. [‡]Department of Pathology and Laboratory Medicine, University of British Columbia, Vancouver, Canada.

[§]Address correspondence to:

Asad Jan, DANDRITE, Department of Biomedicine, Ole Worms Allé 3, DK-8000 Aarhus C, Denmark. Tel. +45 87 15 36 92; Email: ajan@aias.au.dk; ORCID ID: 0000-0002-3636-0070

Contents

- Supplementary Figure Legends, 1-6
- Supplementary Figures, 1-6
- Supplementary Tables, S1-S2

Supplementary Figure Legends

Supplementary Figure 1. Immunostaining of phospho-alpha synuclein (p-aSyn, S129) in lumbar spinal grey matter of M83$^{+\backslash+}$ mice. (A) Representative images showing p-aSyn (S129) IHC in lumbar grey matter of PBS injected cohort- dpi 45 (dpi, days post-injection; DH, dorsal horn; IG, intermediate grey; VH, ventral horn; DWC, dorsal white column; LWC, lateral white column; VWC, ventral white column; 20X magnified views, scale bar=100 µm). Yellow dashed lines outline the boundary between grey and white matter compartments in each image. Primary antibody in Supplementary Figure 1A: p-aSyn (S129)- 11A5. **(B)** Representative images showing dual immunofluorescence detection of p-aSyn (S129, in green) and neuronal nuclei marker (NeuN, in red) in lumbar grey matter of PBS injected cohort- dpi 45 (dpi, days post-injection; DH, dorsal horn; IG, intermediate grey; VH, ventral horn; 20X magnified views, scale bar=100 µm). Compare Supplementary Figure 1A-B with Figure 2A-C (PFF aSyn injected cohorts). Primary antibodies in Supplementary Figure 1B: p-aSyn (S129)- 11A5 and NeuN- A60. **(C)** Representative images showing dual immunofluorescence detection of p62 (Sqstm1, in green) and p-aSyn (S129, in red) in the lumbar ventral horn of PBS injected cohort- dpi 45 and PFF aSyn injected cohorts (dpi, days post-injection; DH, dorsal horn; IG, intermediate grey; VH, ventral horn; 40X magnified views, scale bar=50 µm). Merge images with DAPI (nuclear stain, in blue) are also shown. Yellow arrows in merge images point to instances of double positive cells in VH. Notice also a p-aSyn (S129) positive but p62 negative cell in PFF dpi 21 (White arrow in the merge image). Primary antibodies in Supplementary Figure 1C: p-aSyn (S129)- D1R1R and p62.

Supplementary Figure 2. Immunostaining of phospho-alpha synuclein (S129) in brain regions of M83$^{+\backslash+}$ mice. (A-B) Representative images showing p-aSyn (S129) IHC in the indicated brain regions of PBS injected cohort- dpi 45 (in A) and PFF aSyn injected cohort- dpi 14 (in B). (20X magnified views, scale bar=100 µm; dpi, days post-injection. Compare Supplementary Figure 2B with Figure 3A-B PFF aSyn injected cohorts- dpi 21 and dpi 45. **(C)** Representative images showing p-aSyn (S129) IHC in the indicated brain regions of PFF aSyn injected cohort- dpi 45 (20X magnified views, scale bar=100 µm; dpi, days post-injection; image showing cerebellar nuclei: Lat and Int. indicate lateral and interposed nuclei; image showing striatum: LV. indicates lateral ventricle). Primary antibody in Supplementary Figure 2A-B: p-aSyn (S129)- 11A5.

Supplementary Figure 3. Immunostaining of phospho-alpha synuclein (S129) within brain regions of PFF aSyn injected M83$^{+\backslash+}$ mice, dpi 45. (A) Representative images showing 5X low magnification views (scale bar= 500 µm). Bregma co-ordinates (in mm) are derived from Paxinos and Franklin's 4th edition with the following topographical landmarks: (**Forebrain-1**) Cpu, caudate putamen; M1, primary motor cortex; (**Forebrain-2**) LV, lateral ventricle; CA3, cornu ammonis area 3 of hippocampal formation; DM, dorsomedial hypothalamus; VP, ventralposterior thalamus;

(**Midbrain**) RN, red nucleus; N. III, oculomotor nucleus; PAG, periaqueductal grey; Aq. cerebral aqueduct; (**Pons**) 4V, fourth ventricle; LC, locus coeruleus; VN, vestibular nuclei; Gi, gigantocellular reticular nuclei; IRtA, intermediate reticular nucleus; (**Medulla**) MdV, ventral medullary reticular nucleus). Also see Figure 3A-B. Primary antibody in Supplementary Figure 3A: p-aSyn (S129)- 11A5.

Supplementary Figure 4. Dual immunofluorescence detection of phospho-alpha synuclein (p-aSyn, S129) and astroglial marker GFAP in the lumbar spinal grey matter of M83$^{+\backslash+}$ mice. (A-C) Representative images showing dual immunofluorescence detection of p-aSyn (S129, in green) and astroglial marker Glial fibrillary acidic protein (GFAP, in red) in the ventral and dorsal horn of PFF aSyn injected cohorts- dpi 14 (A), dpi 21 (B) and dpi 45 (C) (dpi, days post-injection; DH, dorsal horn; VH, ventral horn; 40X magnified views, scale bar=50 µm). Merge images with DAPI (nuclear stain, in blue) are also shown. Primary antibodies in Supplementary Figure 4A-C: p-aSyn (S129)- 11A5 and GFAP- D1F4Q.

Supplementary Figure 5. Immunofluorescence co-detection of phospho-alpha synuclein (p-aSyn, S129), astroglial marker GFAP and phagocyte marker CD68 in the select brain regions of M83$^{+\backslash+}$ mice. (A-B) Representative images showing immunofluorescence co-detection of p-aSyn (S129, in green), astroglial marker Glial fibrillary acidic protein (GFAP, in red) and phagocyte marker CD68 (Lamp4, in violet) in PBS (A) or PFF (B) injected cohorts at dpi 45. Regions examined include: midbrain periaqueductural grey-PAG, pontine gigantocellular nuclei-Gi, and pontine vestibular nuclei- VN (4v. indicates the fourth ventricle). (dpi, days post-injection; 20X magnified views, scale bar=100 µm). Merge images with DAPI (nuclear stain, in blue) are also shown. Primary antibodies in Supplementary Figure 5A-B: p-aSyn (S129)- EP1536Y, GFAP-ab4674 and CD68- FA11.

Supplementary Figure 6. Immunostaining of phospho-alpha synuclein (p-aSyn, S129) in post-mortem human midbrain sections. (A-B) Panoramic and 20X magnified views of midbrain sections showing p-aSyn, S129 immunostaining in a control (in A; #ii, Supplementary Figure S2) and a PD case (in B; PD #iv, Supplementary Table S2) in SN and PAG (SN, substantia nigra; PAG, periaqueductal grey; scale bar=100 µm). Also see Figure 5B. Antibody in Supplementary Figure 6A-B: 81A.

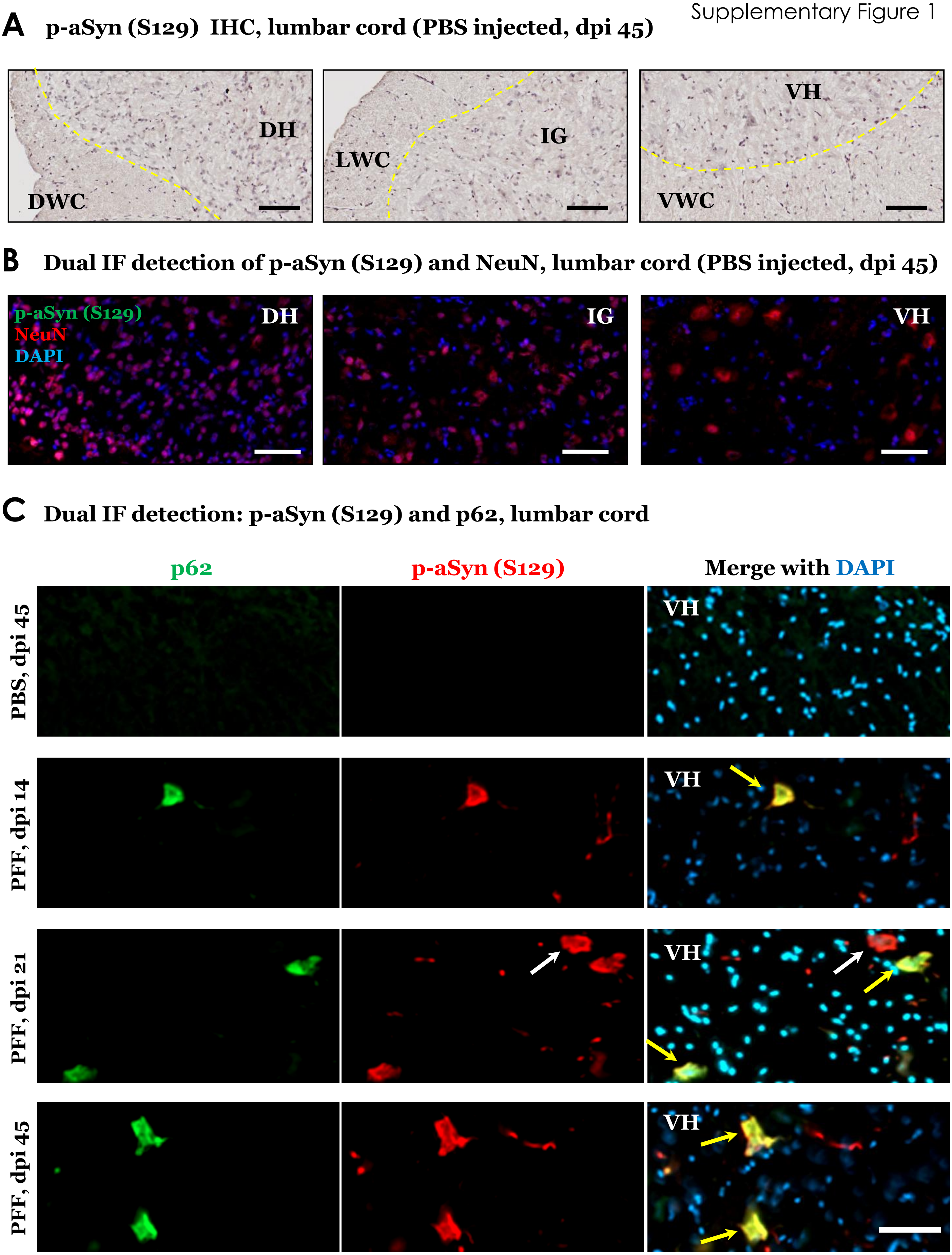
A p-aSyn (S129) IHC, lumbar cord (PBS injected, dpi 45)
DH
DWC
LWC
IG
VH
VWC
B Dual IF detection of p-aSyn (S129) and NeuN, lumbar cord (PBS injected, dpi 45)
p-aSyn (S129)
NeuN
DAPI
DH
IG
VH
C Dual IF detection: p-aSyn (S129) and p62, lumbar cord
p62
p-aSyn (S129)
Merge with DAPI
PBS, dpi 45
PFF, dpi 14
PFF, dpi 21
PFF, dpi 45
VH

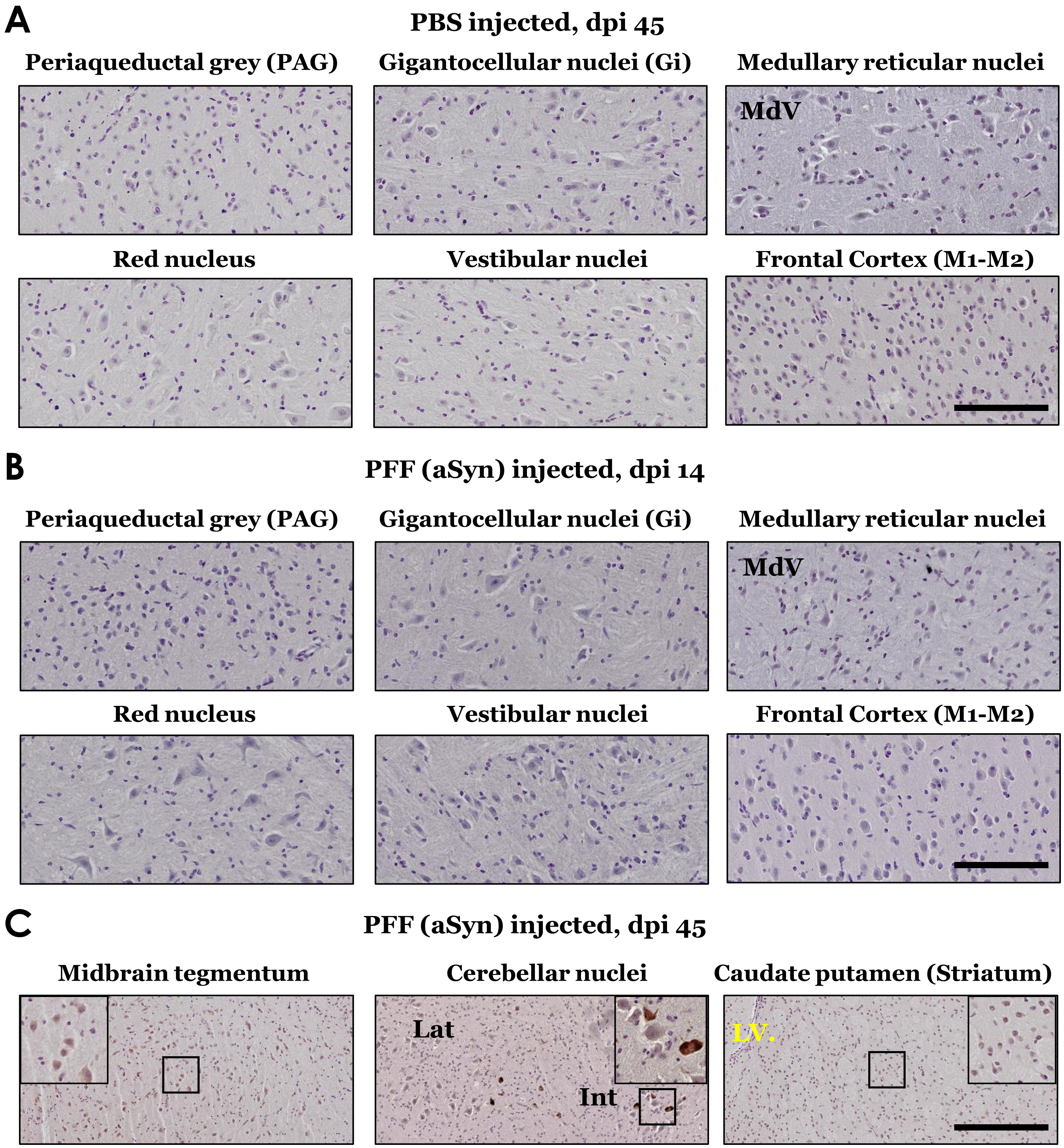
A
PBS injected, dpi 45
Periaqueductal grey (PAG)
Gigantocellular nuclei (Gi)
Medullary reticular nuclei
MdV
Red nucleus
Vestibular nuclei
Frontal Cortex (M1-M2)
B
PFF (aSyn) injected, dpi 14
Periaqueductal grey (PAG)
Gigantocellular nuclei (Gi)
Medullary reticular nuclei
MdV
Red nucleus
Vestibular nuclei
Frontal Cortex (M1-M2)
C
PFF (aSyn) injected, dpi 45
Midbrain tegmentum
Cerebellar nuclei
Lat
Int
Caudate putamen (Striatum)
LV.

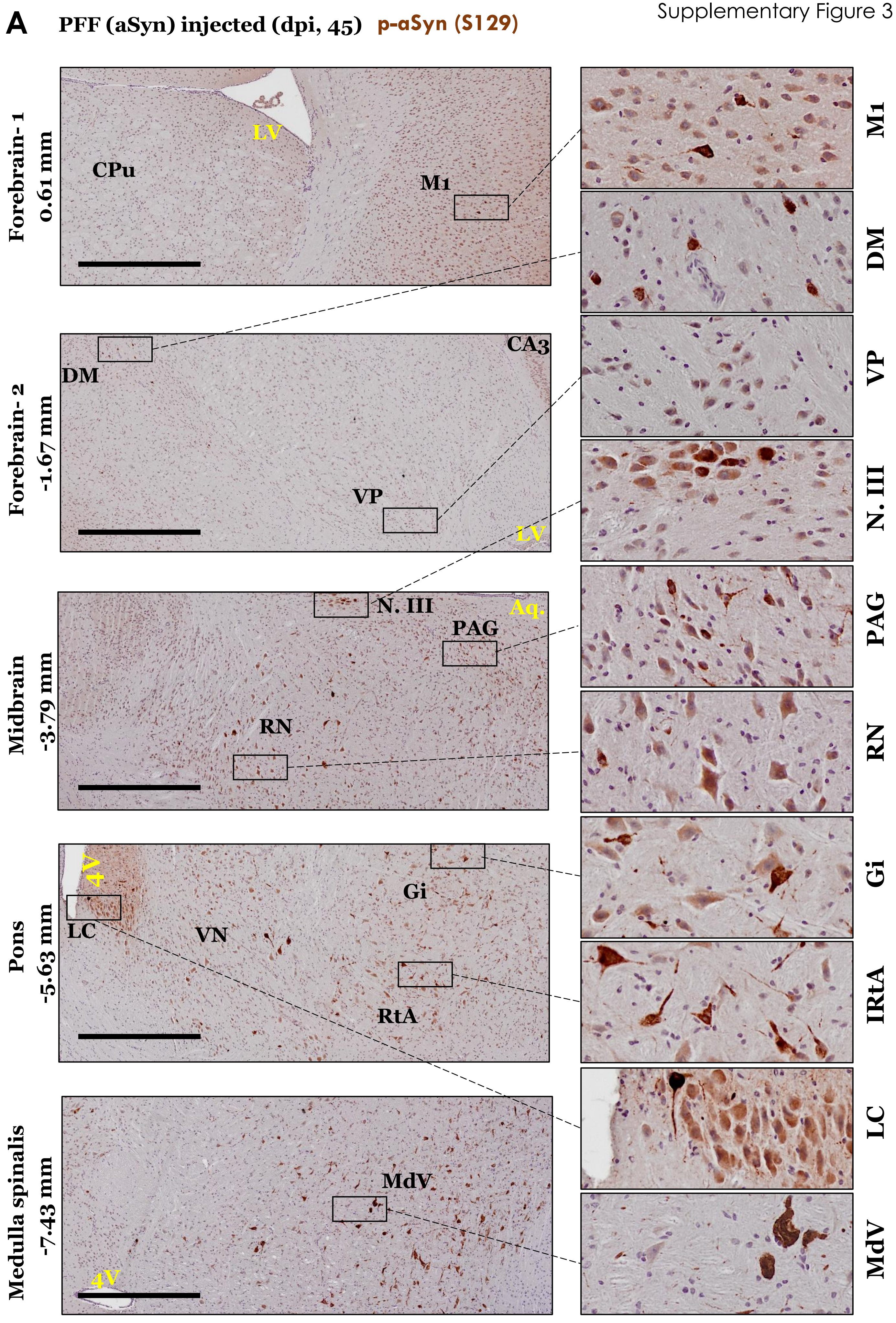
A
PFF (aSyn) injected (dpi, 45)
p-aSyn (S129)
Forebrain- 1
0.61 mm
LV
CPu
M1
DM
CA3
Forebrain- 2
-1.67 mm
VP
LV
N. III
Aq.
PAG
Midbrain
-3.79 mm
RN
4V
Gi
LC
VN
Pons
-5.63 mm
RtA
MdV
Medulla spinalis
-7.43 mm
4V
M1
DM
VP
N. III
PAG
RN
Gi
IRtA
LC
MdV

Dual IF detection: p-aSyn (S129) and GFAP, lumbar cord

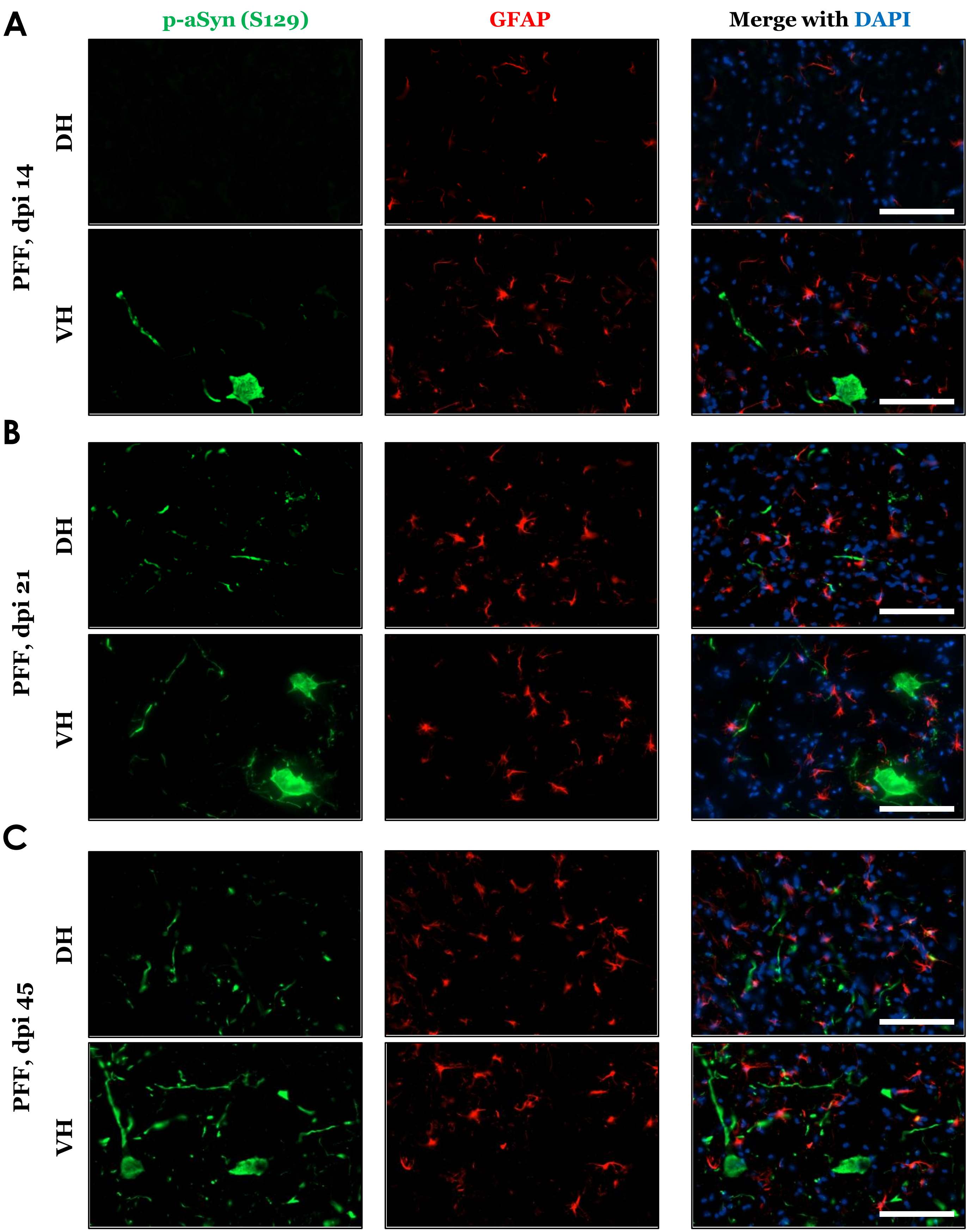

IF detection: p-aSyn (S129), GFAP and CD68

A **PBS, dpi 45**

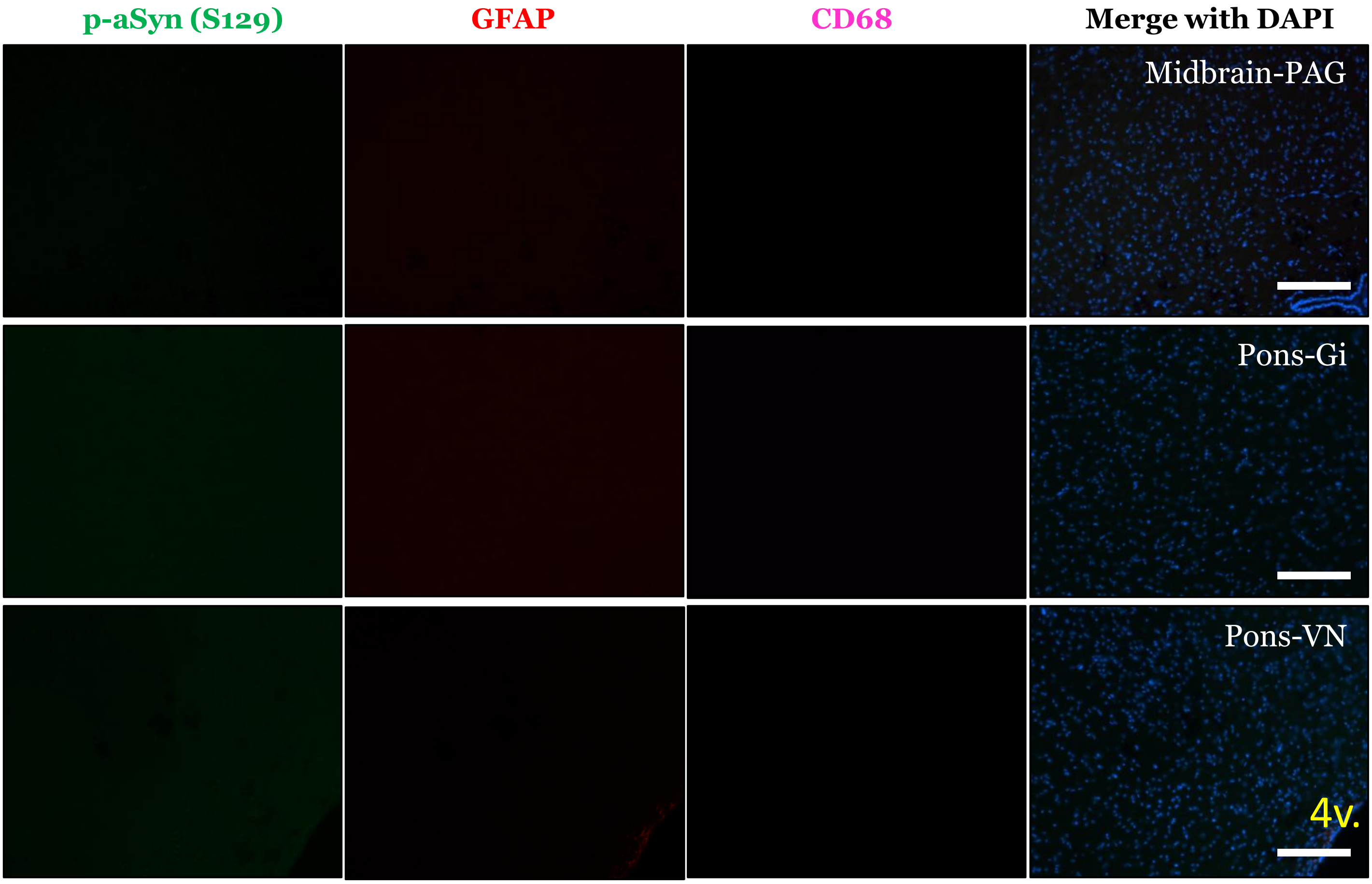

B **PFF, dpi 45**

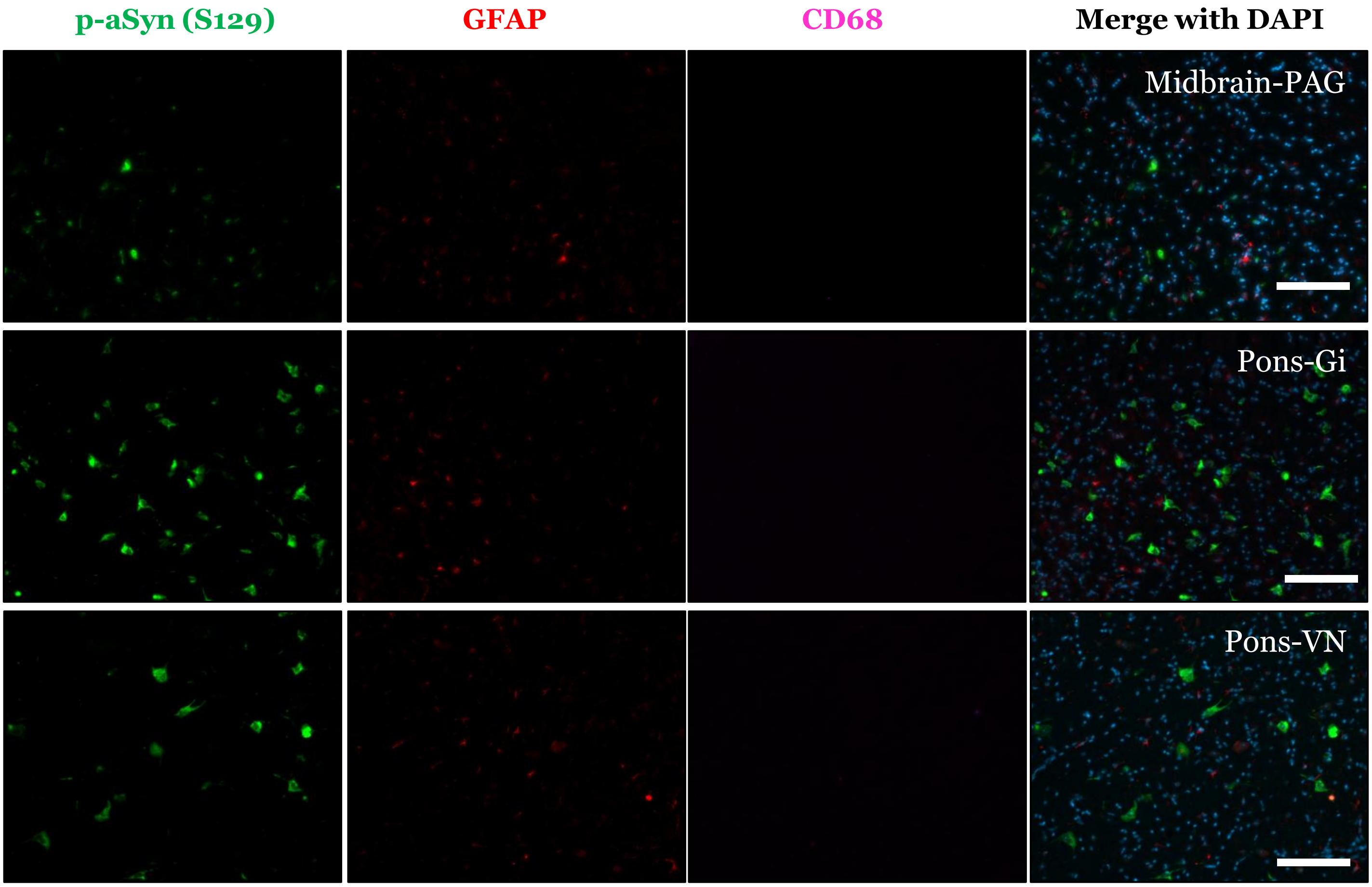

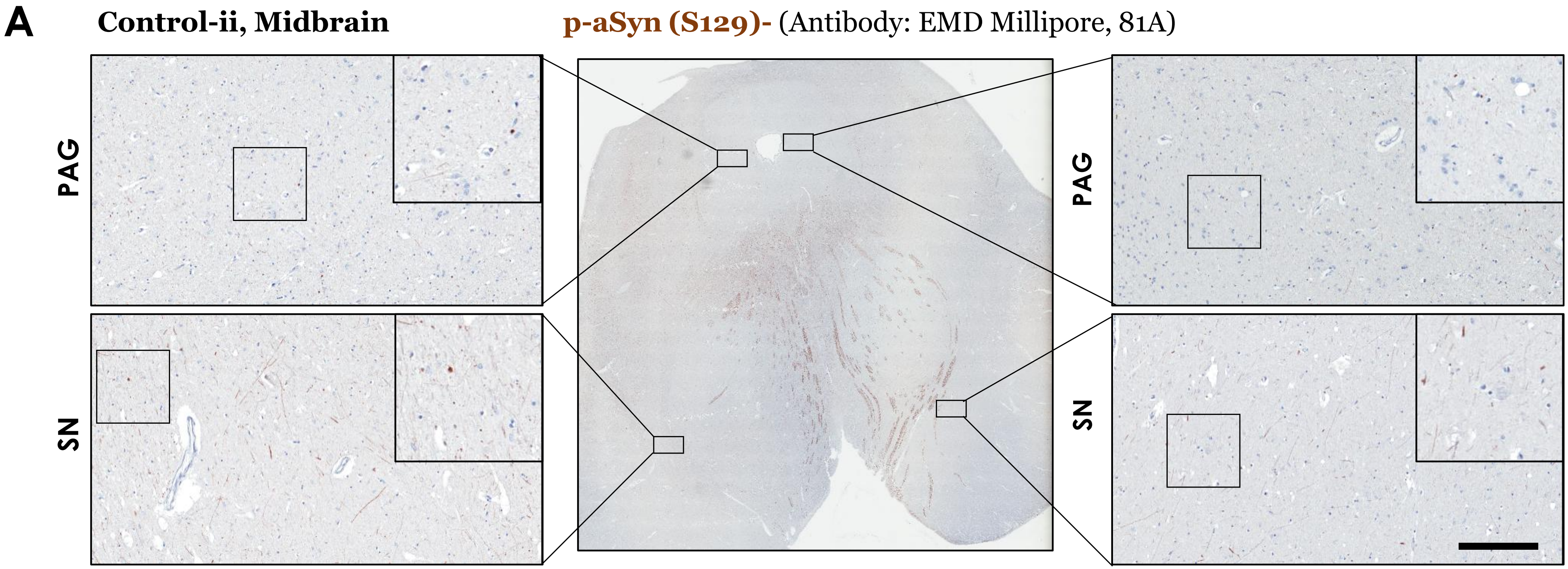
A
Control-ii, Midbrain
p-aSyn (S129)- (Antibody: EMD Millipore, 81A)
PAG
SN
PAG
SN

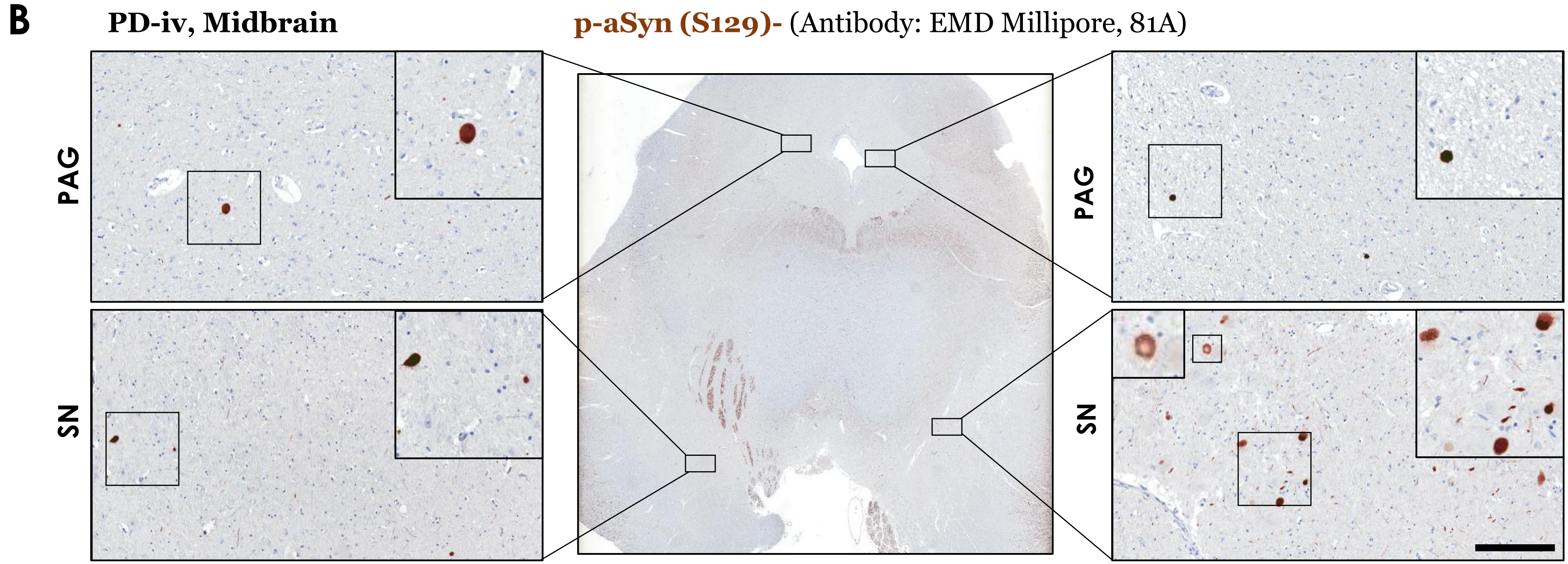
B
PD-iv, Midbrain
p-aSyn (S129)- (Antibody: EMD Millipore, 81A)
PAG
SN
PAG
SN

Supplementary Table S1. Demographic and pathological variables

	**Control**	**PD**	**MSA-P**	**MSA-C**
Gender (M/F)	2/1	2/1	4/2	5/1
Disease duration (y)	-	19±15	6±2	5±3
Age at death (y)	77±10	71±9	70±9	75±7
Postmortem delay (h)	19±13	17±16	19±15	9±5
Braak Lewy body stage (/6)	-	5±1	-	-
Braak neuritic stage (/6)	0.9±1.5	0.4±0.8	0.8±2.0	0±0
CERAD plaque score (/3)	0.2±0.6	0.3±0.8	0±0	0±0

Cases: PD, Parkinson disease; **MSA-P**, multiple system atrophy parkinsonian subtype; **MSA-C**, multiple system atrophy cerebellar subtype.

Supplementary Table S2. Demographics, Control and PD cases

Case ID	Gender	Age (years)	
**Controls**			
Control-i	Male	52	
Control-ii	Female	66	
**PD**			*Additional postmortem pathology*
PD-i	Male	69	Limbic predominant DLB
PD-ii	Male	81	Cortical DLB
PD-iii	Male	74	Rare lewy bodies in limbic and neocortex
PD-iv	Female	68	Cortical DLB
